# Rapid Detection of Rare Deleterious Variants by Next Generation Sequencing with Optional Microarray SNP Genotype Data

**DOI:** 10.1002/humu.22818

**Published:** 2015-07-22

**Authors:** Christopher M. Watson, Laura A. Crinnion, Juliana Gurgel‐Gianetti, Sally M. Harrison, Catherine Daly, Agne Antanavicuite, Carolina Lascelles, Alexander F. Markham, Sergio D. J. Pena, David T. Bonthron, Ian M. Carr

**Affiliations:** ^1^ School of Medicine, University of LeedsLeedsUnited Kingdom; ^2^ Yorkshire Regional Genetics ServiceSt James's University HospitalLeedsUnited Kingdom; ^3^ Department of PediatricsFaculty of Medicine, Universidade Federal de Minas GeraisBelo HorizonteBrazil; ^4^ Laboratory of Clinical GenomicsUniversidade Federal de Minas GeraisBelo HorizonteBrazil; ^5^ GENE—Nucleo de Genetica Medica de Minas GeraisBelo HorizonteBrazil

**Keywords:** exome, next generation sequencing, autozygosity mapping, software

## Abstract

Autozygosity mapping is a powerful technique for the identification of rare, autosomal recessive, disease‐causing genes. The ease with which this category of disease gene can be identified has greatly increased through the availability of genome‐wide SNP genotyping microarrays and subsequently of exome sequencing. Although these methods have simplified the generation of experimental data, its analysis, particularly when disparate data types must be integrated, remains time consuming. Moreover, the huge volume of sequence variant data generated from next generation sequencing experiments opens up the possibility of using these data instead of microarray genotype data to identify disease loci. To allow these two types of data to be used in an integrated fashion, we have developed *AgileVCFMapper*, a program that performs both the mapping of disease loci by SNP genotyping and the analysis of potentially deleterious variants using exome sequence variant data, in a single step. This method does not require microarray SNP genotype data, although analysis with a combination of microarray and exome genotype data enables more precise delineation of disease loci, due to superior marker density and distribution.

## Introduction

Autozygosity mapping using individuals from complex consanguineous families [Lander and Botstein, 1987; Mueller and Bishop, 1993] has been used to localize the genes causing many rare, autosomal recessive diseases. Initially, the pace at which autozygous regions could be delineated was a significant hurdle for new mapping projects. However, the development of high‐density genome‐wide SNP‐genotyping microarray reagents rendered this step comparatively simple, once suitable analysis packages had been developed to scrutinize the data [Carr et al., 2006, 2009; Seelow et al., 2009]. Similarly, the identification of possibly deleterious sequence variants was formerly a major undertaking. Once again, with the advent of exome sequencing, the practical aspects of variant detection became facile, while bioinformatic analysis became more complex. The latter required computer programs that could both detect sequence variants and then filter them against a number of specified parameters, including position, predicted effect, quality score, and genotype [Li et al., 2009; McKenna et al., 2010; Watson et al., 2014].

While the huge information content of genome‐ or exome‐wide sequence variant data has complicated its analysis, it has also opened up the possibility of analyzing the sequence data itself to map disease loci, without the use of SNP microarray genotypes [Alkuraya, 2013; Carr et al., 2013; Görmez et al., 2013]. An important additional opportunity afforded by exome data is that parent and sibling data can be directly inspected to identify possibly deleterious de novo mutations [Pagnamenta et al., 2012; Blue et al., 2014].

There are a number of practical difficulties in using exome variant data rather than SNP microarray genotypes to map a disease locus [Carr et al., 2013]. For instance, the exome data may have higher genotyping error rates (especially for variants within duplicated regions), and may show poor coverage of regions with low gene densities. These issues do not typically affect microarray SNP genotype datasets; these generally have many more verified variants distributed evenly across the genome, making it possible to define the extent of a disease locus more accurately. The sites of locus‐defining recombination events can be more accurately mapped using high‐density SNP microarray data than is possible with exome sequence data, especially when recombination occurs in regions of low gene density. Some of these limitations can be abrogated by the use of variant data from whole genome (rather than exome) sequencing (WGS). The falling cost of WGS will eventually make this more commonplace. However, substantial read depths are required to permit reliable SNP genotyping from WGS, so that at today's prices, affordable (if imperfect) mapping information is more readily obtained by SNP genotyping and exome sequencing.

Given the practical advantages of combining exome variant data with microarray SNP genotype data to identify disease genes, we developed *AgileVCFMapper*. This program uses NGS variant datasets, with or without microarray SNP genotype data, to aid the characterization of disease loci and the rapid identification of the correct candidate deleterious variant(s). The program creates a variant dataset of the exome‐derived variants from all individuals in the analysis. Read depth data for each patient can also be imported and used to distinguish homozygous reference positions from those where read depths are too low for accurate genotyping.

*AgileVCFMapper* can then identify variants in shared autozygous regions or de novo mutations. Alternatively, for nonautozygous pedigrees, the variant dataset can be exported to allow its analysis by other genetic mapping software. In the former case, *AgileVCFMapper* can mirror the analyses performed by *AutoSNPa* [Carr et al., 2006] or *IBDFinder* [Carr et al., 2009] to identify shared regions of autozygosity (with or without a common disease haplotype). The analysis can be extended by importing microarray SNP genotype data, so that regions of autozygosity defined by exome and microarray SNP genotype datasets can be integrated. Since the exome data may contain the actual disease‐causing variant, it is then possible to interactively view variants in a region on a per‐gene basis. This feature is particularly useful when analyzing conditions with either a known or candidate disease gene; in such cases, the rapid identification of deleterious variants in these genes can save a significant amount of effort by negating the need for further variant screening and filtering. This program, along with an extensive user guide, can be downloaded from http://dna.leeds.ac.uk/agile/AgileVCFMapper/.

## Methods

### Library Preparation, Sequencing, and Variant Identification

Genomic DNA (3 μg) was first sheared into 200‐ to 300‐bp fragments using a Covaris S2 sonicator (Covaris, Inc., Woburn, MA) and then purified using a QiaQuick column (Qiagen, Chatsworth, CA). Illumina‐compatible sequencing libraries were prepared using Agilent library preparation reagents as per the manufacturer's instructions. A five‐cycle enrichment PCR was used to generate the libraries, which were captured using the Agilent v5 exome reagent hybridization probe set followed by ten cycles of posthybridization enrichment PCR. Paired‐end, 100‐bp reads were generated using a HiSeq 2500 in high‐output mode with five samples pooled per lane. The sequence data were aligned to the human genome (hg19) using BWA [Li and Durbin, 2009] and variants identified using GATKlite [McKenna et al., 2010]. Nucleotide numbering uses +1 as the A of the ATG translation initiation codon in the reference sequence, with the initiation codon as codon 1. Similarly, amino acid numbering uses +1 at the initiating methionine residue of the reference sequence.

### Microarray SNP Genotype Data Requirements

Genotyping should be performed using very high‐density SNP microarrays, such as the Affymetrix SNP 5.0, SNP 6.0, or Axiom chips. If genotype data from Illumina microarrays are used, the files must first be annotated using the appropriate *Illumina2Affy* conversion program. These accessory programs are available from http://dna.leeds.ac.uk/illumina2affy/.

### Subjects

Ethical approval for this project was obtained from South Yorkshire Research Ethics Committee (Ethics ID: 11/H1310/1).

*Pedigree 1*: It consisted of a consanguineous nuclear family with two offspring both affected by mitochondrial DNA depletion syndrome‐5 (MIM# 612073), due to a homozygous mutation in the *SUCLA2* gene (NM_003850.2:c.998A>G, NM_003850.2(SUCLA2_i001):p.(Asp333Gly)), for which both parents were heterozygous carriers. While the parents were first cousins, there is no other history of consanguinity in the pedigree. Exome variant data were available for both affected siblings and their parents. This variant has been previously reported as pathogenic [Matilainen et al., 2015] and has been submitted to the *SUCLA2* variant database at http://databases.lovd.nl/shared/genes/SUCLA2.

*Pedigree 2*: It consisted of a highly consanguineous family affected by primary ciliary dyskinesia (CILD19; MIM# 614935) caused by a homozygous mutation in *LRRC6* (NM_012472.4(LRRC6_v001):c.630del, NM_012472.4(LRRC6_i001):p.(Trp210Cysfs*12)). Exome variant data were only available for two affected siblings, while Affymetrix SNP 6.0 microarray SNP genotype data were available for a third affected sibling and two unaffected siblings. This pedigree was described previously [Watson et al., 2014], and the variant was submitted to the *LRRC6* gene variant database at http://databases.lovd.nl/shared/genes/LRRC6.

*Pedigree 3*: It consisted of the nonconsanguineous NIGMS CF1038 nuclear family (http://ccr.coriell.org/sections/collections/nigms/ExtendedFamilies.aspx?PgId=52). Affymetrix SNP 6.0 genotype data and exome variant data were available for both parents and four siblings, of whom two have cystic fibrosis (MIM# 219700) (Supp. Table S1).

*Pedigree 4*: To demonstrate the ability to identify de novo mutations, the data file for patient NA07383 from pedigree 3 was modified to include rs28931614:G>A as a heterozygous variant in *FGFR3*. This variant causes achondroplasia (MIM# 100800) and is one of the most frequently observed recurrent de novo germline mutations in the human genome (Bellus et al., 1995).

### Identification of Autozygous Regions in Exome Sequence Variant Data

Exome variant datasets can be generated using a wide range of aligners and variant callers, with an almost unlimited number of analysis parameter combinations. Consequently, before autozygous regions can be identified using *AgileVCFMapper*, it is necessary to standardize the variant calling. Irrespective of the genotype given in the VCF file, *AgileVCFMapper* redetermines each variant's genotype, according to the following rules:

1. If >80% of reads match the reference sequence, the genotype is set to homozygous reference (AA).
2. If >80% of reads suggest the same alternative (nonreference) sequence, the genotype is set to homozygous variant (BB).
3. If 40‒60% of reads identifies the same variant sequence, the genotype is called as heterozygous (AB).
4. If the variant's genotype is not identified by any of the previous steps, it is flagged as a “no call.”

The minimum read depth used to identify autozygous regions is independent of the minimum read depth value used to display variants. It varies depending on whether an “rs” number is linked to the variant in the VCF file. If a variant has an rs number, the minimum read depth is set to 15 reads for homozygous and 30 reads for heterozygous genotypes, respectively. If the variant has no rs number, the minimum read depth is increased to 75 and 150 reads for homozygous and heterozygous positions, respectively. Once the genotypes have been reassigned, runs of homozygous variants are identified. If two consecutive homozygous regions, containing more than 25 variants each, are interrupted by a single heterozygous variant, the heterozygous variant's genotype is reassigned as “No call” and the homozygous runs are recalculated. Again, heterozygous variants interrupting homozygous runs are discounted. This process is reiterated four times before the final list of homozygous runs is fixed and used to identify autozygous regions over 500 kb in length.

### Identification of Autozygous Regions in Microarray SNP Genotype Data

Since microarray genotype calling is more consistent than exome variant genotyping, it is not necessary to regenotype the microarray data. However, it is still necessary to detect and discount aberrantly called heterozygous positions interrupting autozygous regions. This is performed in the same manner as described for exome data, except that the minimum length of homozygous runs that can be interrupted by a single heterozygous variant is set to a value calculated by multiplying the number of SNPs on the microarray by 7 × 10^−4^. Although this effectively limits the analysis to microarrays with more than 10,000 genotypes, it is not a significant problem, since most microarray genotyping chips produced since 2007 are more complex than this.

### Implementation

#### Importing variant data into AgileVCFMapper

*AgileVCFMapper* is primarily aimed at identifying deleterious variants in exome data; while the analysis can incorporate microarray SNP genotype data, the use of the latter is optional. The exome data should be formatted as a standard VCF file, and include the reference and variant allele read depths in the optional information columns. The exome data are used to create a unified dataset of all the variants in the analysis. However, since individual exome data files typically only contain data for heterozygous and homozygous variant (nonreference) alleles, the internal variant dataset may lack read depth data for positions where an individual is either homozygous reference or has low read depth at that position. While it is not essential to add this missing read depth data for all possible analysis methods, it is particularly useful when identifying de novo mutations or exporting the unified dataset to be analyzed by other programs such as *Phaser* [Carr et al., 2012], as described below. Read depth data are imported using data formatted as *GATKLite* read depth files. All the data files should be placed in an empty folder with the variant data files having a *.vcf extension. The read depth files have the same names as the corresponding variant files, but with a *.txt extension. Once the exome variant data, read depth data, and the minimum read depth at which variants are retained have been selected, the unified variant dataset is created. Once created, *AgileVCFMapper* displays the “analysis methods” window, which allows the selection of each of the analysis method pipelines.

#### Exporting genotype data as a unified variant dataset

**Figure 1:**
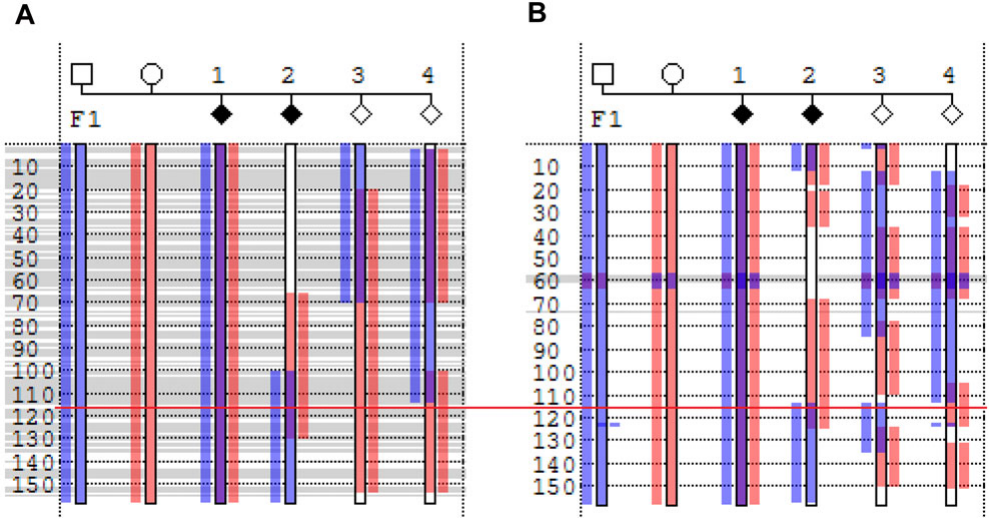
Mapping of the *CFTR* locus in a nonconsanguineous CEPH family (Pedigree 3, see the section Methods), using variant data derived either (**A**) from exome sequencing or (**B**) from microarray SNP genotyping (Affymetrix SNP 6.0). Display of Chromosome 7 haplotypes was performed using *Phaser* [Carr et al., 2012]. In this display, blue and pink vertical bars denote the haplotypes inherited by the first affected offspring from the father and mother, respectively. The disease locus must be located in a region where all affected individuals share the same combination of maternal (pink) and paternal (blue) haplotypes as this first individual. This is denoted by a purple color in the central vertical bar (region of overlap between the affected paternal and maternal haplotypes). Unaffected offspring should be discordant with their affected siblings at the disease locus (i.e., no purple bar). In this example, the actual position of the *CFTR* gene is marked by a horizontal red line. It can be seen that the candidate region containing *CFTR* is considerably smaller in (**B**), due to the superior resolution and distribution of the microarray SNPs.

Once the data files have been imported, the variant data can be exported as a unified dataset, which can then be used by other mapping programs. However, if the optional read depth data are not imported, this dataset will contain a large number of “no call” genotypes at positions which are homozygous for the reference sequence in an individual, and so missing from the variant file. Since an excess of “no call” genotypes may severely limit the value of the dataset for any subsequent scrutiny, it is strongly recommended to include the optional read depth data files in the analysis. Figure 1 shows a comparison of the mapping of the cystic fibrosis locus by *Phaser* with exome data (Fig. 1A) or Affymetrix SNP 6.0 microarray genotype data (Fig. 1B), for Pedigree 3. Both methods identify the correct locus. However, the lower number of variants in the exome data, and their uneven distribution, results in a larger disease interval than when using microarray data. This is mirrored in the analysis of the other autosomes (Supp. Fig. S1); the regions of common descent tend to appear larger in the exome data than in the comparable microarray data.

#### Analyzing variant data with AgileVCFMapper

*AgileVCFMapper* allows the visualization of exome data and the detection of possible recessive disease‐causing variants in consanguineous individuals. It can also identify de novo mutations.

#### Analyzing data from consanguineous patients

*AgileVCFMapper* extends the methods of both *AutoSNPa* and *IBDFinder* by allowing the analysis to be performed on exome sequence data. By selecting the “Consanguineous” analysis method, the user is prompted to identify the disease status for each of the data files. *AgileVCFMapper* then identifies the locations of autozygous regions, as described above in the section “Identification of autozygous regions in exome sequence variant data.” Autozygous regions from affected and unaffected individuals are highlighted as pale blue or pink rectangles, respectively.

#### Identifying concordant and nonconcordant autozygous regions

**Figure 2:**
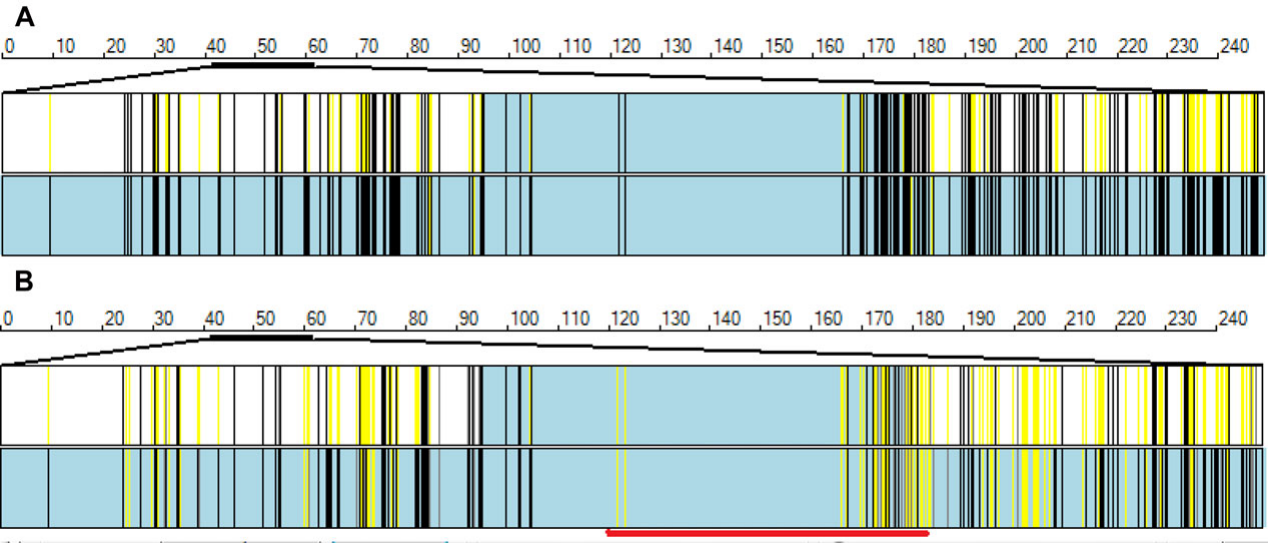
Autozygosity mapping using exome variant data. The blue regions indicate the extent of autozygous regions deduced by *AgileVCFMapper*. The black and yellow vertical lines indicate the genotypes of variants as described in the section “Identifying concordant and nonconcordant autozygous regions.” **A**: The option “autozygous regions” has been selected; **B**: The option “common regions” has been chosen; consequently, in (**B**) some variants that are homozygous but nonconcordant between the two individuals under study have changed from black to yellow. The red horizontal bar indicates a region of overlapping but nonconcordant autozygosity.

Figure 2A and B shows the visualization of the two nonconcordant overlapping autozygous regions in Pedigree 1 (not at the disease locus). The “autozygous regions” display option mirrors the corresponding function of *IBDFinder*, with black and yellow vertical lines indicating the positions of homozygous and heterozygous variants, respectively. This enables autozygous regions from individuals with no disease haplotype in common to be scanned for shared regions of autozygosity (Fig. 1A). Alternatively, when patients are believed to share the same disease haplotype, the “common regions” option should be used. Note that because of the limitations of image resolution, if a position on the image represents data for multiple variants, that position is marked by a black or a yellow line if all the genotypes are concordant homozygous or all heterozygous, respectively. However, for positions that contain nonconcordant genotypes, the line will be yellow, orange, or black if <80%, 80–90%, or >90% of the genotypes are concordant for the same homozygous genotype. In this way, nonconcordant regions are easily identified and discounted. This option requires the presence of homozygous reference variant genotypes and so only functions correctly when the analysis includes the optional read depth data.

#### Including microarray SNP genotype data in the analysis

**Figure 3:**
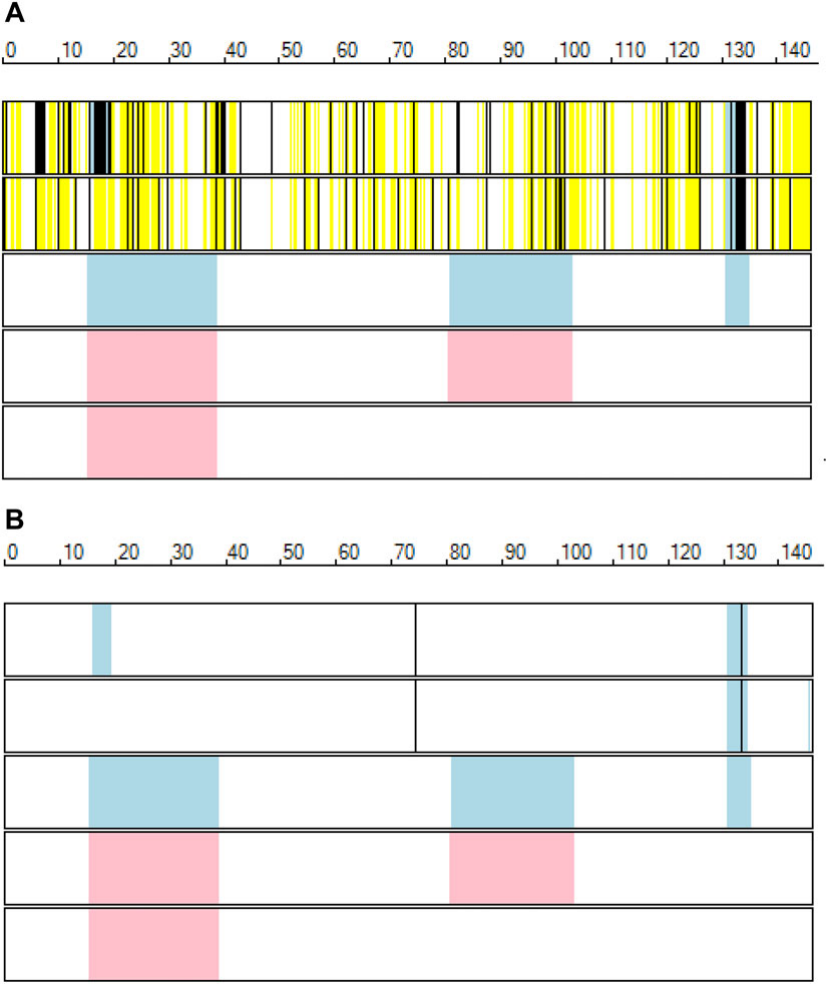
Visualization of Chromosome 8 exome (upper two rows) and microarray genotype (lower three rows) data for five siblings from Pedigree 2. The blue and pink rectangles indicate the positions of autozygous regions in affected and unaffected individuals, respectively. **A**: The black and yellow vertical lines identify homozygous and heterozygous exome positions, respectively. In contrast, in (**B**) more restrictive display settings are selected; black vertical lines identify exome variants that are homozygous in both affected individuals for which exome data are available and have no associated rs number in the original VCF variant data files. The variant at approximately 133.5 Mb, lying within the region of shared autozygosity, is a frameshift mutation in the *LRRC6* gene and is thought to be the disease‐causing variant.

It is possible to view the exome data in the context of autozygous regions identified in other individuals using microarray SNP genotype data, rather than exome data. However, since exome and microarray datasets contain very few variants in common, it is not possible to identify haplotypes in common between exome and microarray genotype data. Consequently, only autozygous regions are displayed for microarray data. Again, these are shown as blue or pink rectangles, representing regions in affected and unaffected individuals, respectively (Fig. 3). Supp. Figure S2 shows a comparison of autozygous regions identified by *AgileVCFMapper* using exome versus microarray genotype data.

#### Identifying possible disease‐causing variants

In contrast to mapping disease loci using microarray SNP genotype data, when using exome data it is possible or probable that the disease‐causing variant itself is present in the variant dataset. Accordingly, *AgileVCFMapper* can filter the dataset for variants, which have only the homozygous variant genotype in affected individuals, by selecting the “affected genotypes” option. If the data are annotated with rs numbers, it is also possible to filter the variants further by excluding those that have an rs number (using the “affected genotypes without RS ID” option). This can be a powerful filtering technique if unaffected close relatives of the patients are included in the analysis (Fig. 3).

#### Identification of genotypes showing non‐Mendelian inheritance

If the dataset consists of data from a nuclear family, with both parents and at least one affected offspring, it is possible to screen the data for de novo mutations, which result in either loss or gain of heterozygosity. Since single‐base de novo mutations predominantly occur at positions that are homozygous for the reference sequence allele in both parents, they can be identified as heterozygous genotypes in affected offspring at positions that are homozygous reference sequence in both the parents and unaffected siblings. By selecting the “show heterozygous variants only” option, variants whose genotype suggests this mode of inheritance will be highlighted, with the genotypes shown as blue, red, black, or gray lines indicating homozygous reference sequence, homozygous variant sequence, heterozygous variants, and uncalled genotype, respectively. Similarly, de novo or previously unidentified deletions can be identified by the presence of unexpected homozygous genotypes, which can be viewed by selecting the “show homozygous variants only” option, with genotypes color coded as described above.

#### Examination of the variants in conjunction with gene sequence data

**Figure 4:**
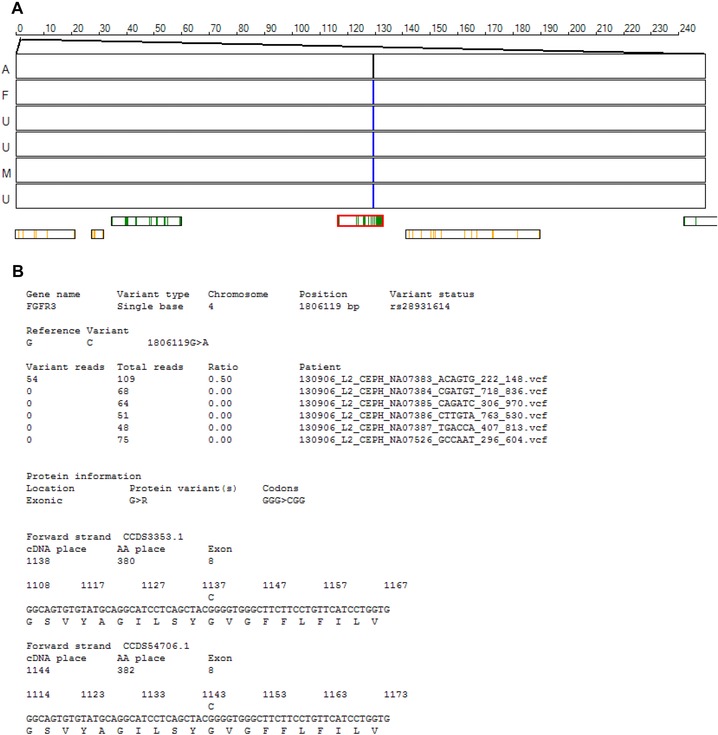
**A**: Visualization of a simulated heterozygous de novo mutation in *FGFR3*, introduced in silico into the VCF data file of one patient NA07383 of Pedigree 3 (NIGMS CF1038). This individual is marked “A” to the left, the other three siblings as “U.” The parents are designated “F” and “M.” The heterozygous de novo variant appears as a black vertical bar; the homozygous reference genotype in all other individuals is indicated in blue. **B**: The displayed meta‐information for the variant describes its location in the gene, its effect on the protein's sequence (if any), and the read depth information for each individual in the analysis.

An increasingly important step in novel disease gene identification will be the detection of individuals with deleterious mutations in known disease genes. To allow the rapid screening of individuals for deleterious variants in such genes, *AgileVCFMapper* can rapidly examine variants in them. This analysis is performed by entering a file containing sequence data and the exon coordinates for all the genes, in either a CCDS dataset [Pruitt et al., 2009] or the RefSeq dataset [Pruitt et al., 2014]. This *.GAF file can be created using AgileGAFCreator (http://dna.leeds.ac.uk/agile/AgileGAFCreator/). Once these data have been imported, each variant in a gene may be viewed with respect to its effect on the resultant protein sequence, possible exon splicing, and read depths for each individual (Fig. 4). When viewing variant data that have been filtered (i.e., the “show heterozygous variants only,” “show homozygous variants only,” “affected genotypes,” or “affected genotypes without RS ID” options), the currently displayed variants are selected, while for other visualization options it is possible to select a gene by name and then iterate through a list of variants in that gene. This allows variants in known disease genes to be rapidly selected and screened as possible deleterious variants.

## Results

To illustrate the functionality of *AgileVCFMapper*, the program was used to analyze exome data from Pedigrees 1, 2, and 3.

Since the Pedigree 1 data contained exome data for two affected siblings and both of their parents, it was analyzed by first identifying variants that were homozygous in affected individuals, but not in their parents. This excluded 99.4% of the variants in the unified variant dataset, with only 822 variants retained. This number was reduced to just 24 when variants with an rs number were removed, and of these, only 11 variants were found to lie within autozygous regions. Finally, the locations of the remaining variants were observed relative to the positions of exon sequences, with only four variants directly affecting a protein's sequence. A subsequent literature review of the genes containing these variants identified *SUCLA2* as the probable causative disease gene, since it had previously been identified as a cause of mitochondrial DNA depletion syndrome‐5, with which the patients had been diagnosed.

In Pedigree 2, the lack of exome data for unaffected relatives of the two affected siblings prevents filtering variants by disregarding those that are homozygous in unaffected individuals. However, 77% (31392) of variants could be discounted just by selecting those that are homozygous in both affected siblings. By screening for homozygous variants in the regions of common autozygosity, the number of possible deleterious variants was reduced to 115. Since microarray SNP genotype data were available for a third affected and two unaffected siblings, it was possible to refine the disease locus to a single region on Chromosome 8 (130.7–134.5 Mb) that contained just 49 variants in 10 genes. By using *AgileVCFMapper* to observe the variants’ positions in each gene, it was determined that only 12 affected an encoded protein sequence. Of the involved genes, it was found that *LRRC6* had previously been identified as causing primary ciliary dyskinesia and so was the most likely disease gene. *LRRC6* was homozygous for a frame shift mutation, which resulted in the loss of the final 256 amino acids, strongly suggesting that this would disrupt the protein's function. Also this variant was the only one in this region that did not possess an rs number, indicating it to be a rare variant.

The analysis of Pedigree 3 was completed using both *AgileVCFMapper*, to create and export a unified variant dataset, and *Phaser* [Carr et al., 2012], an external mapping application, to define the locus using the exported variant data. Since both exome and microarray SNP genotype data were available for all the individuals in this analysis, it was possible to compare the mapping results obtained using the two types of data with *Phaser* (Fig. 1 and Supp. Fig. S1). Blue bars indicate sequences inherited from the father by the first affected child, and pink bars those inherited from the mother by the first affected child. Consequently, at the disease locus all the affected children (and none of their unaffected siblings) should display a blue and pink bar. As can be seen in Figure 1, the *CFTR* gene (marked by the horizontal red line) lies within such a region. It should be noted that the extent of the region of shared alleles tends to be greater when determined using exome data (Fig. 1A) than microarray SNP genotype data (Fig. 1B). This is a function of both the uneven distribution and lower number of variants in the exome dataset, when compared to the microarray data.

Finally, an analysis of Pedigree 4 data (a modified Pedigree 3 dataset, containing the common achondroplasia variant rs28931614:G>A) was performed using the “show heterozygous variants only” option. This identified 38 possible de novo mutations, of which only one was not linked to an rs identifier. (Due to the high incidence of activating de novo mutations in *FGFR3*, several disease‐causing variants have been assigned rs identifiers, so that in this instance filtering variants by the absence of an rs identifier is not appropriate.)

## Discussion

Exome sequencing is a powerful method for the detection of deleterious variants. However, due to the volume of data generated by each experiment, identifying a disease variant can be very difficult without secondary information. This might consist of a list of known or functional candidate disease genes, a disease locus, or a particular pattern of inheritance.

While a list of known disease genes must be collated before the analysis, it is possible to both extract autozygosity mapping information and screen the disease variants simultaneously with *AgileVCFMapper*. In contrast, analysis of out‐bred individuals may require a more complex multistep pipeline, involving the creation of a unified variant dataset, mapping analysis of the dataset, and finally filtering the variants based on the mapping information. While these variant‐filtering approaches appear to differ significantly from one another, they nonetheless include steps common to each pipeline, for example, the creation of a unified variant dataset and distinguishing between those positions with homozygous reference genotypes and those with too few reads to genotype. For this reason, we developed *AgileVCFMapper*, a program that can combine variant data from different individuals to create a unified variant dataset. This dataset can then be either interrogated using *AgileVCFMapper* (to identify de novo mutations, perform autozygosity mapping, and view variants in genes of interest), or exported in a format suitable for analysis by other mapping programs.

Identifying deleterious mutations within an exome variant dataset can be very difficult without inspecting the context in which a variant occurs. Consequently, *AgileVCFMapper* allows the interactive viewing of variant data with reference to a mutation's position in a putative disease locus and/or location within and effect on a candidate disease gene. This feature is particularly useful when searching for variants in known disease genes in order to reach a molecular diagnosis, or when prescreening subjects for variants in known genes before attempting to identify novel disease genes in genetically heterogeneous conditions.

While *AgileVCFMapper* can export its unified variant dataset for analysis by external mapping applications, it can also perform autozygosity mapping analysis on the exome data itself. Irrespective of the method used to identify a disease locus, exome‐derived variant datasets are smaller and not as evenly distributed across the genome as those from a typical SNP microarray. Consequently, exome data do not allow such fine disease locus mapping as microarray genotype data (Supp. Figs. S1 and S2), though it may still be very useful. To bridge the gap between using exome and microarray data, *AgileVCFMapper* can identify and display autozygous regions derived from both data types, although it is not possible to identify common haplotypes within such mixed datasets. This ability to simultaneously visualize autozygous regions from exome and microarray data can allow very productive compromises. For example, a disease locus may be mapped using microarray data from most subjects while exome variant information is gathered from a minority of them. This can be seen in the analysis of Pedigrees 1 and 2, in which the initial variant datasets were rapidly reduced to a very low number of candidate variants, which could easily be examined on an individual basis.

In summary, as with all disease gene mapping methodologies, the ease with which deleterious variants are found using *AgileVCFMapper* is very dependent on the pedigree under analysis. While exome data are far from ideal for gene mapping, it can still be very productively used for this purpose, especially in conjunction with supplementary information of the types mentioned above. The detection of de novo mutations requires a different approach, but again, the ability of *AgileVCFMapper* to identify and display these variants contextually in the genes within which they occur allows each variant to be rapidly screened for a possible deleterious effect. We expect *AgileVCFMapper* to be useful both in the detection of novel disease genes and for the rapid screening of pedigrees for deleterious variants in known disease genes.

*Disclosure statement*: The authors declare no conflict of interest.

## Supporting information

Disclaimer: Supplementary materials have been peer‐reviewed but not copyedited.

**Figure S1**. A comparison between exome‐ and microarray‐derived variant data, displayed by *Phaser* (Carr et al. 2012) for the purpose of mapping a disease locus using a non‐consanguineous pedigree.
**Figure S2**. A comparison between exome‐ and microarray‐derived variant data used to identify autozygous regions in consanguineous individuals.
**Table S1**. The NGMS ids for the individuals in Pedigree 3
