# Supplementary material for: Rapid Detection of Rare Deleterious Variants by Next Generation Sequencing with Optional Microarray SNP Genotype Data
Source: Hum Mutat. 2015 Jul 22;36(9):823–30. doi: 10.1002/humu.22818 (PMC4744743; doi:10.1002/humu.22818)

Supp. Table S1. The NGMS ids for the individuals in Pedigree 3

Individual ID	NIGMs ID
Farther	NA07384
Mother	NA07387
Affected sib (1)	NA07383
Affected sib (2)	NA07526
Unaffected sib (3)	NA07385
Unaffected sib (4)	NA07386

The number in the brackets corresponds to the numbers in Figure 1 and Supp. Figure S1.

Supp. Figure S1. (next page) A comparison between exome- and microarray-derived variant data, displayed by *Phaser* (Carr et al. 2012) for the purpose of mapping a disease locus using a non-consanguineous pedigree. Each pair of images below shows the maternal and paternal origins of a chromosome in four siblings, using data derived from exome variants (left-hand panels) or Affymetrix SNP 6.0 microarray genotype data (on the right). While the two data sets give similar outcomes, there are notable differences between them. These are the result of the lower number of variants, more uneven coverage and higher miscall rate of the data derived from the exomes.

Supp. Figure S1

Chromosome 1

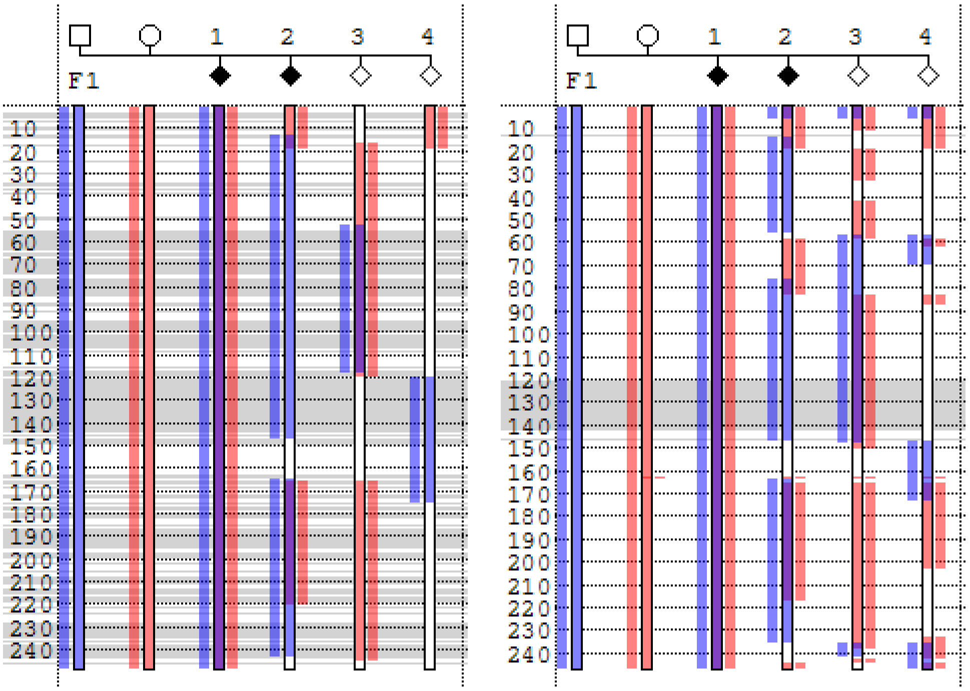

Chromosome 2

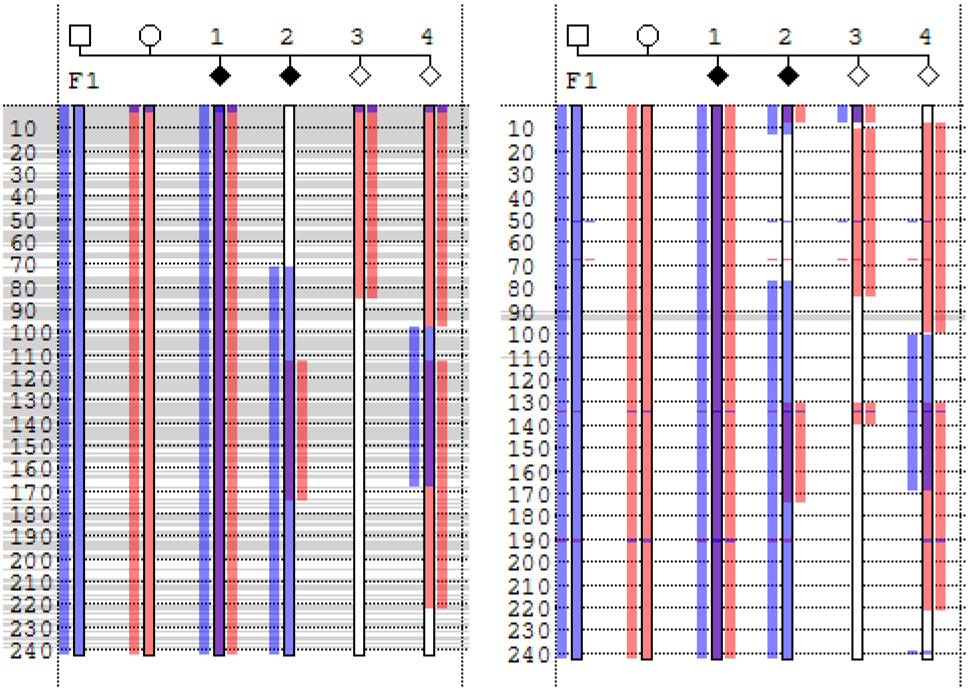

Chromosome 3

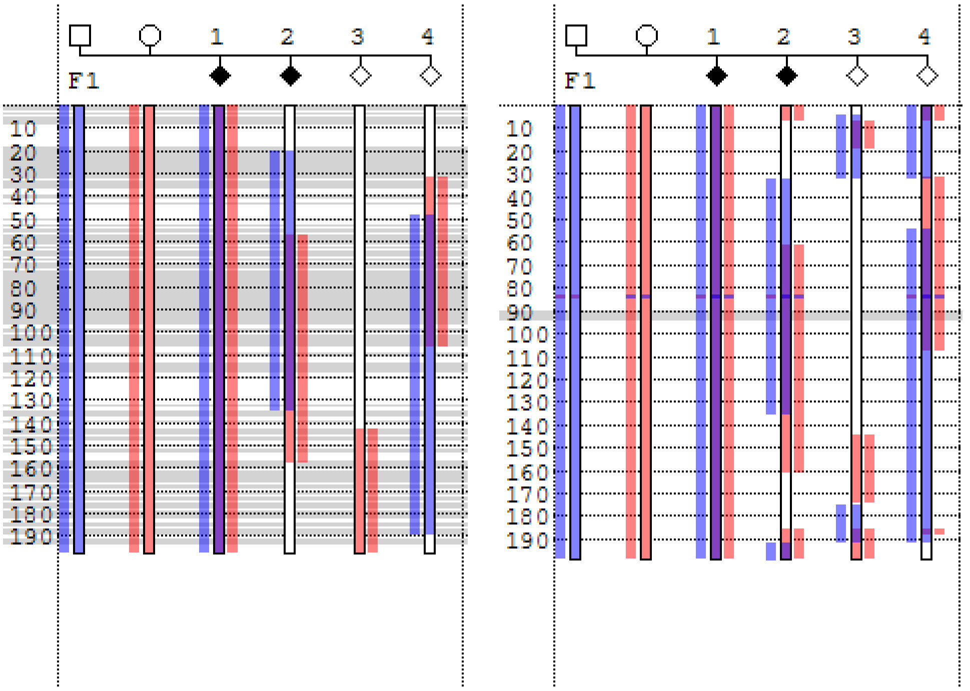

Chromosome 4

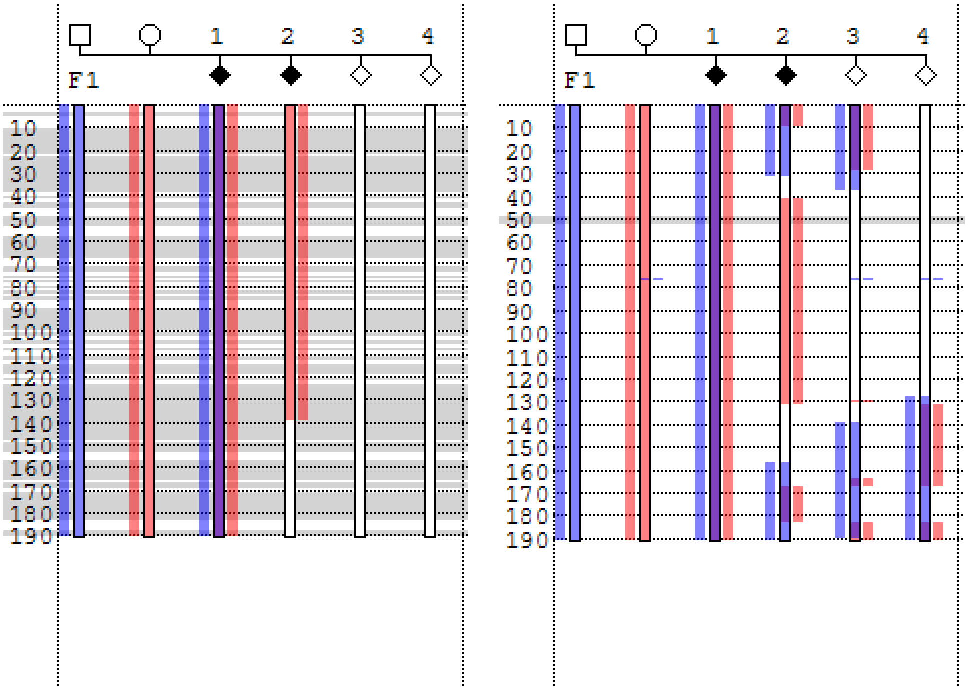

Chromosome 5

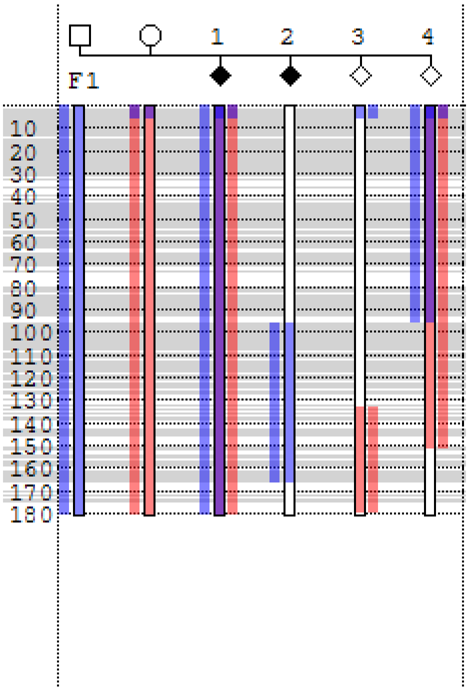

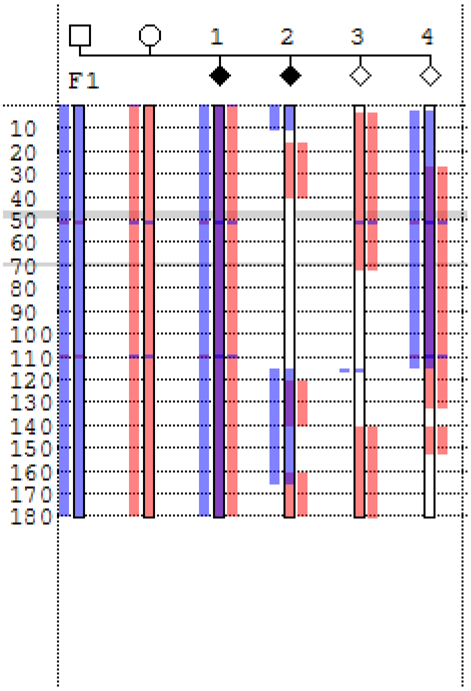

Chromosome 6

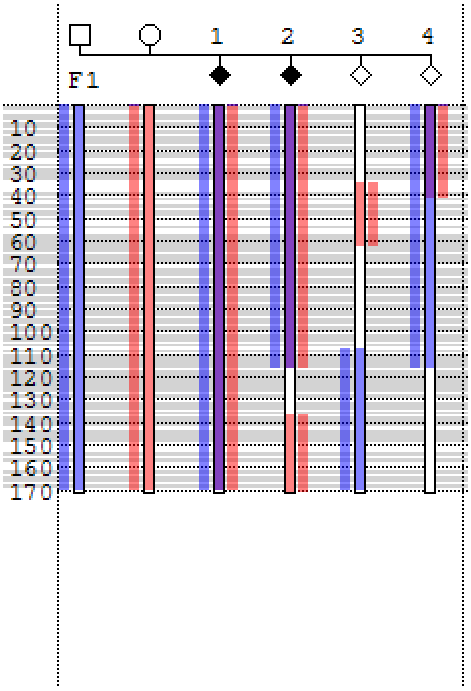

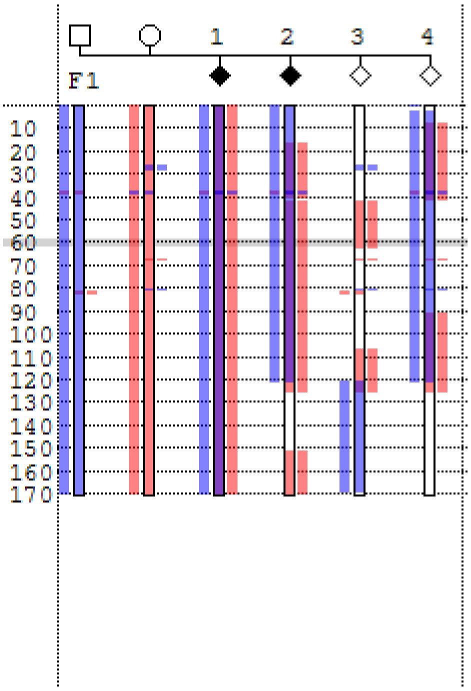

Chromosome 7

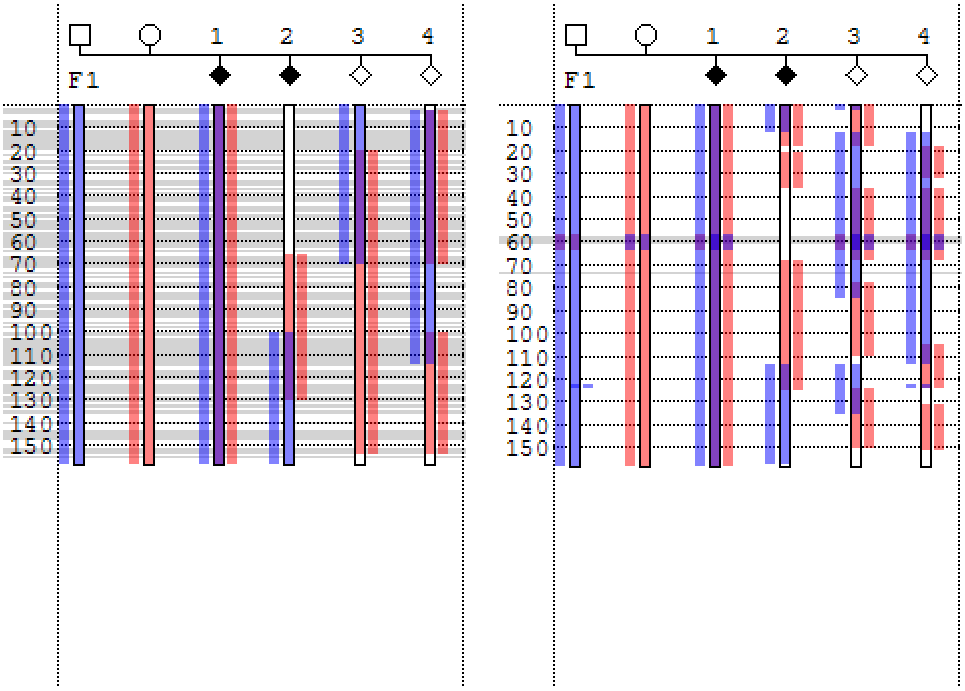

Chromosome 8

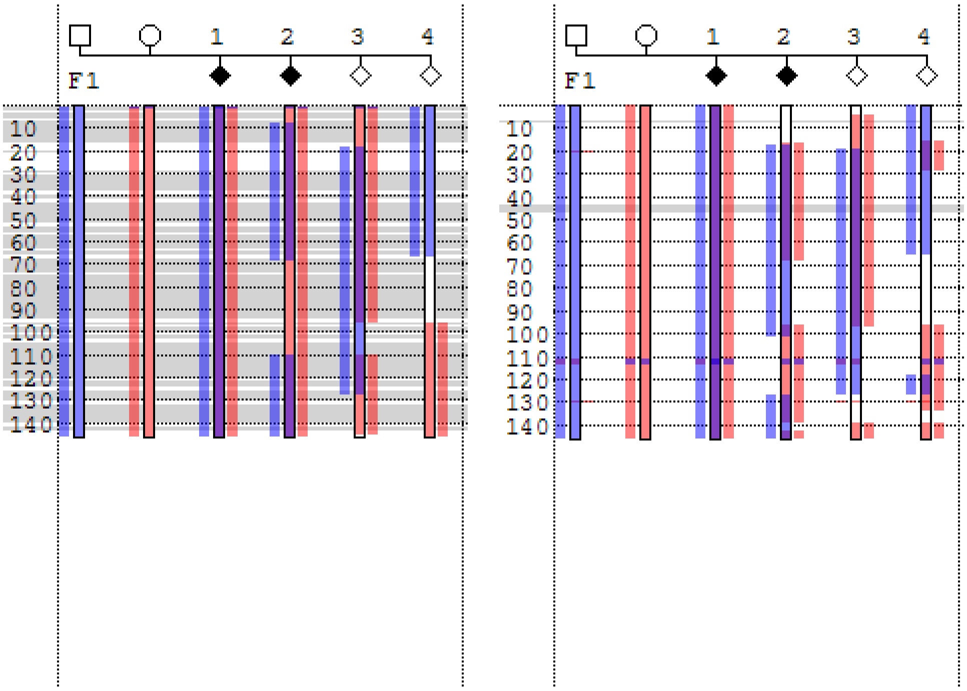

Chromosome 9

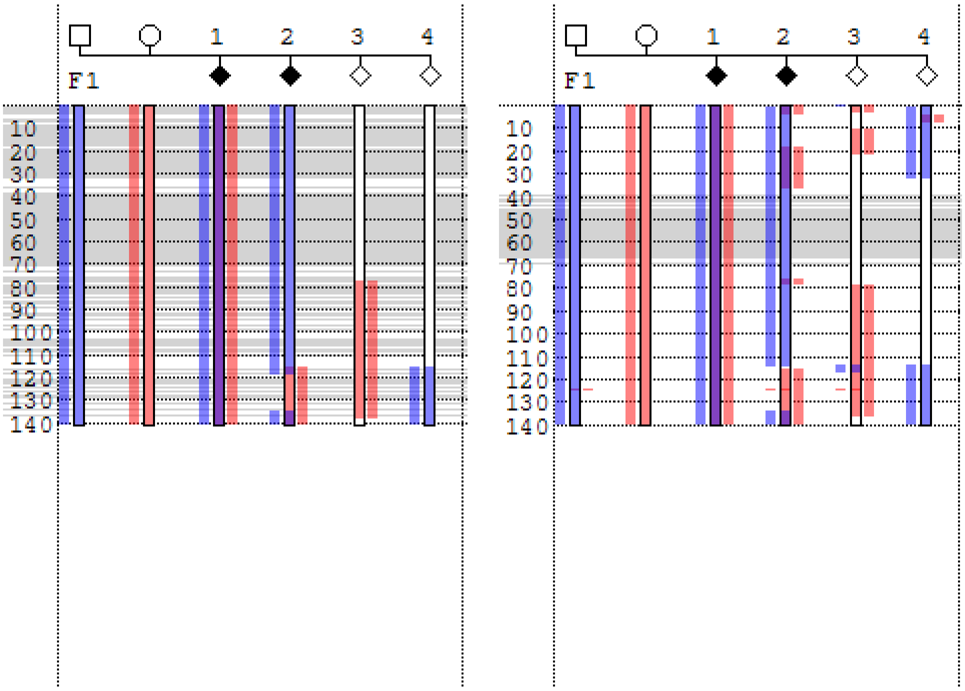

Chromosome 10

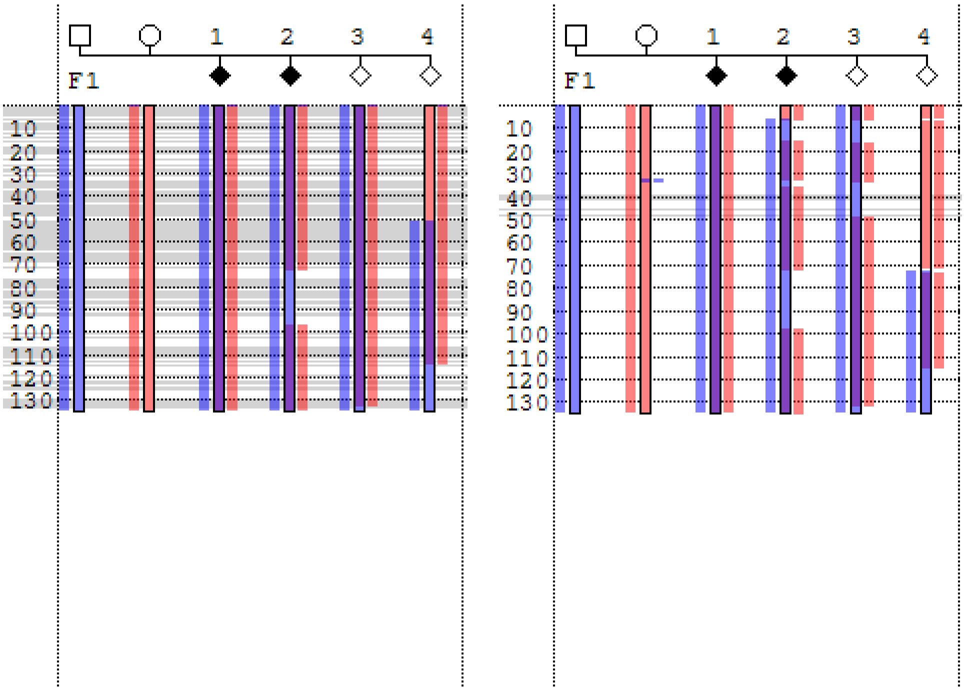

Chromosome 11

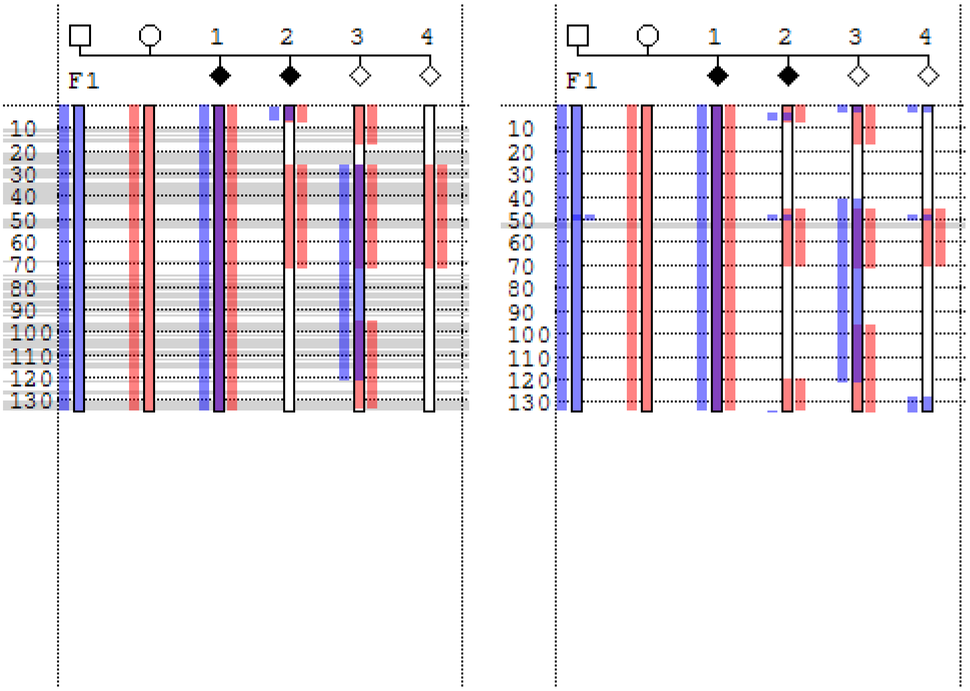

Chromosome 12

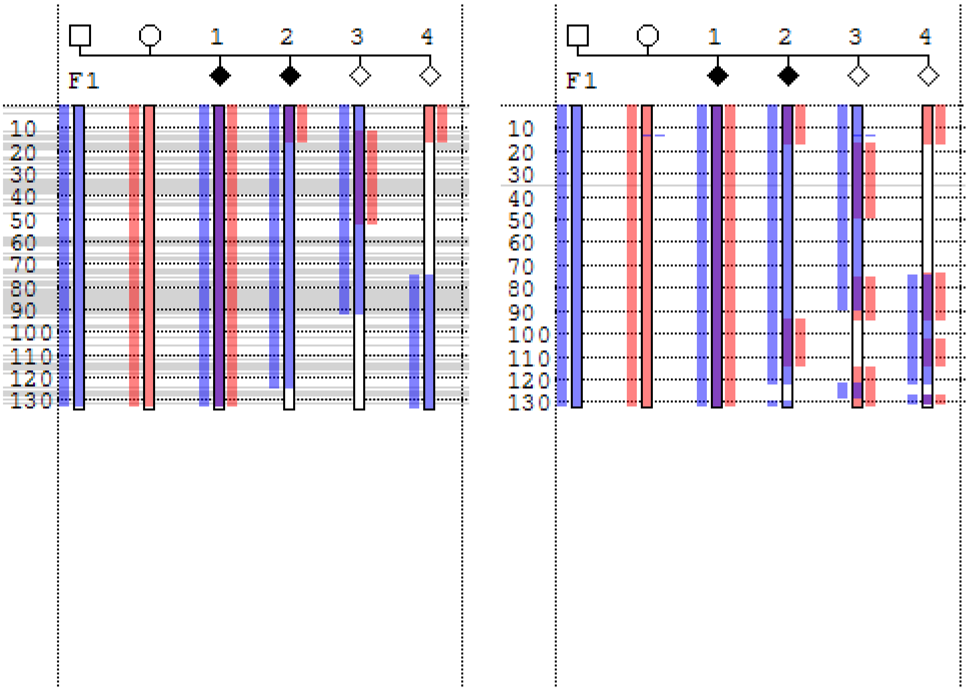

Chromosome 13

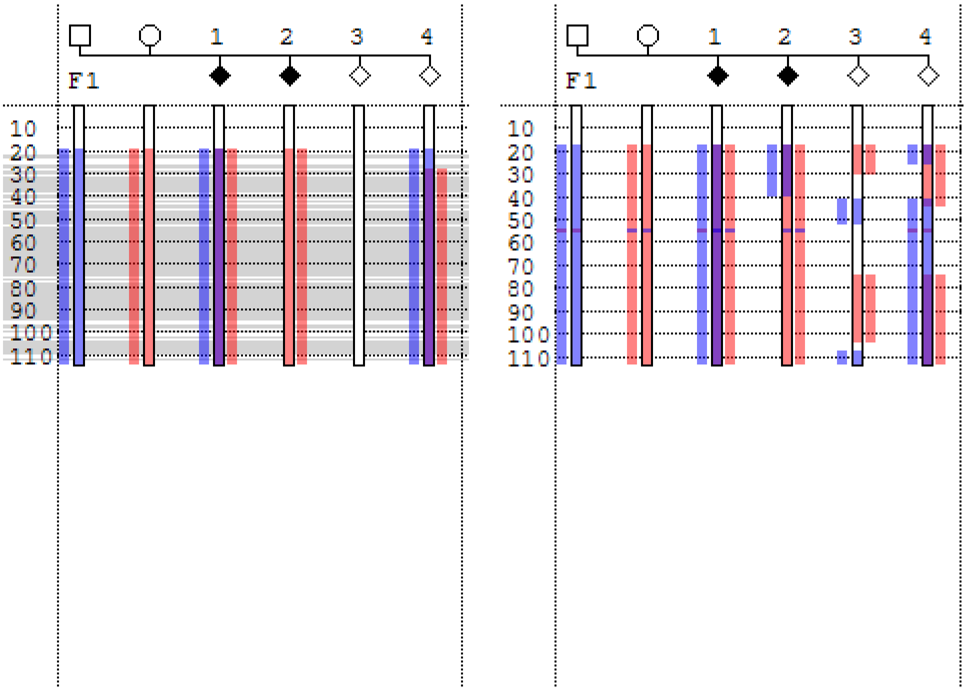

Chromosome 14

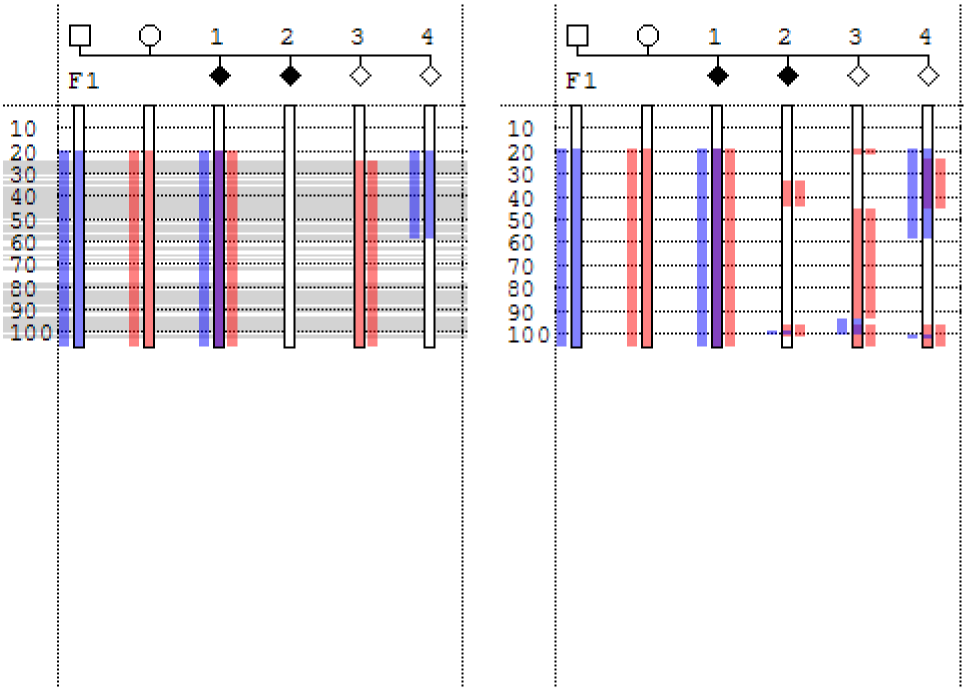

Chromosome 15

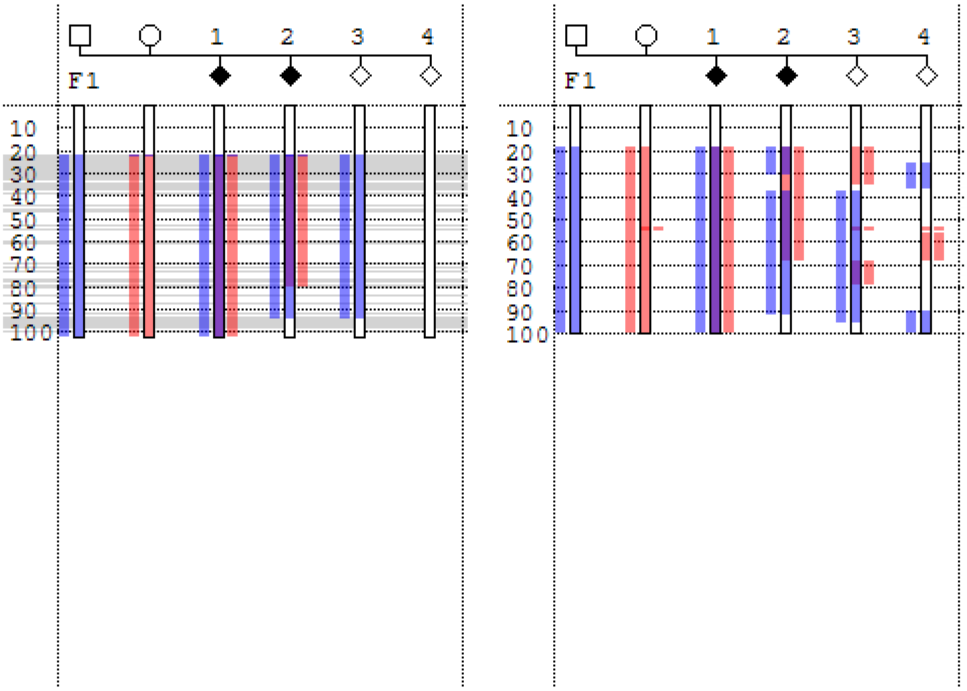

Chromosome 16

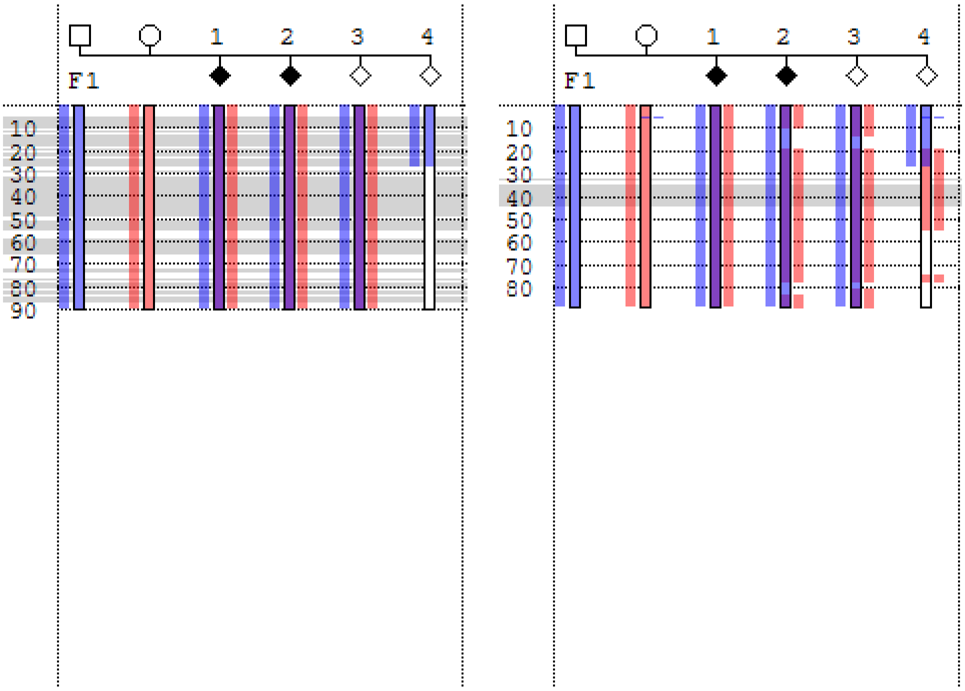

Chromosome 17

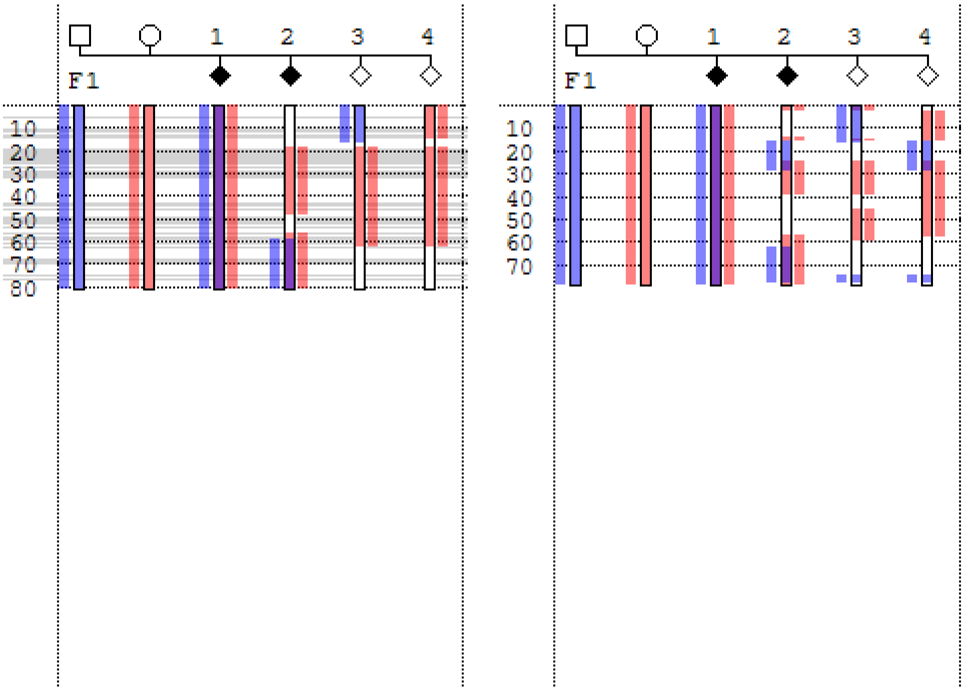

Chromosome 18

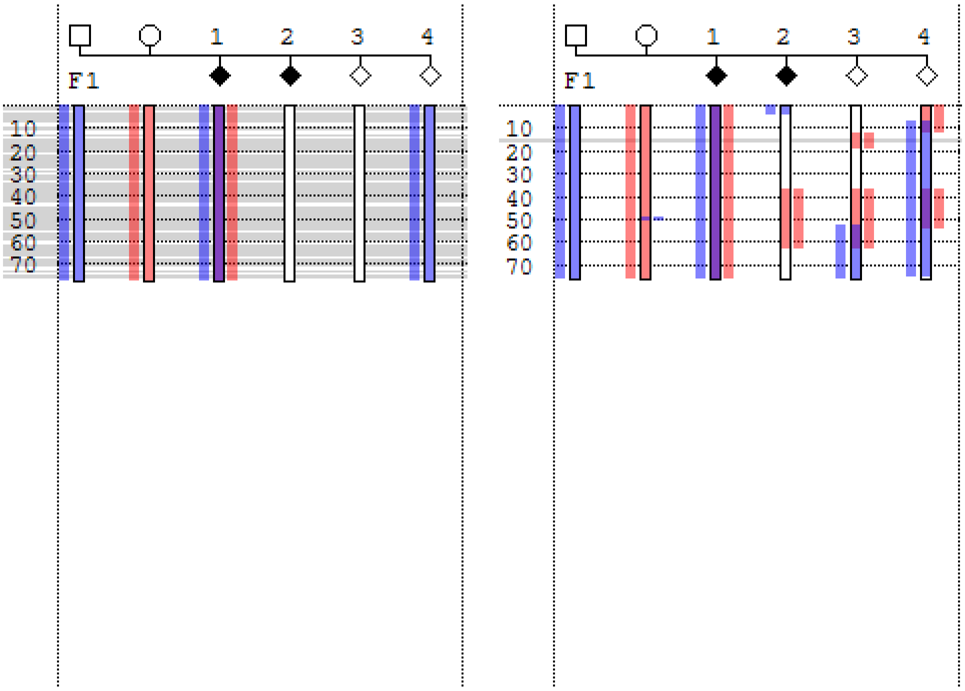

Chromosome 19

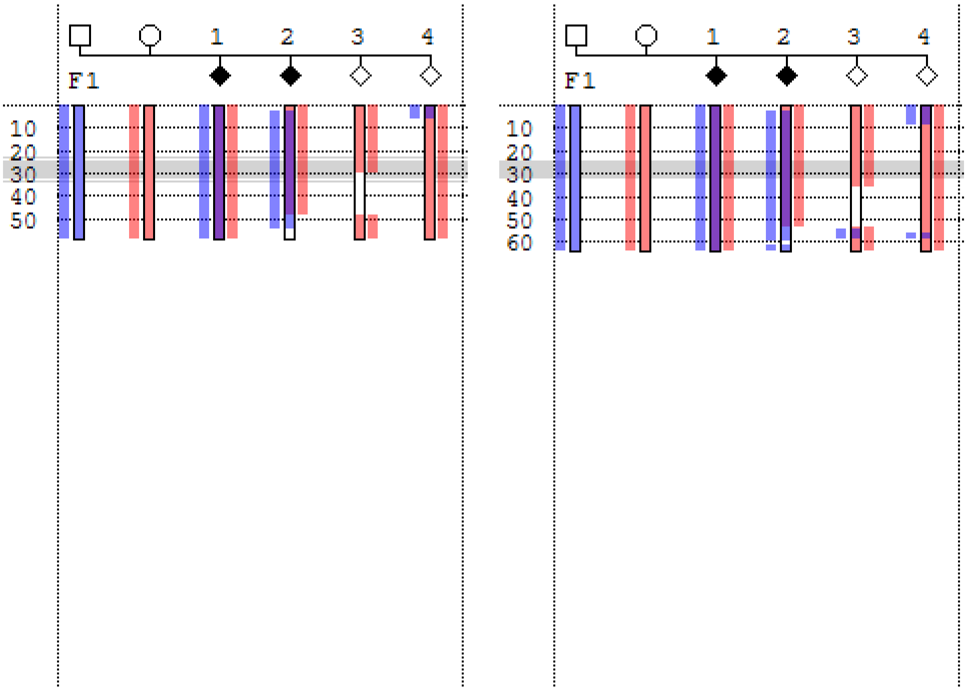

Chromosome 20

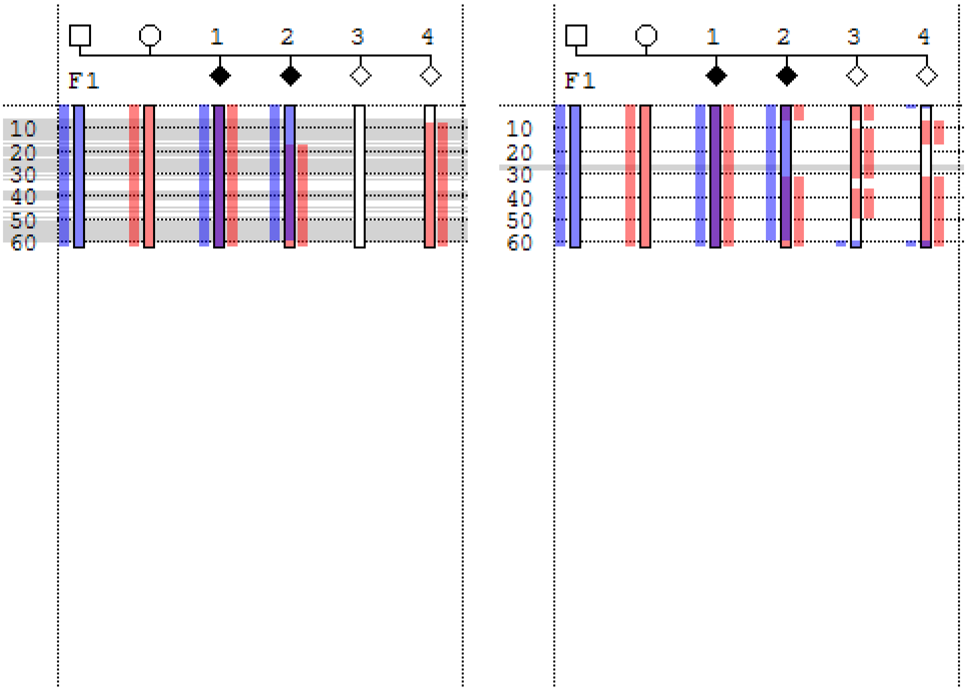

Chromosome 21

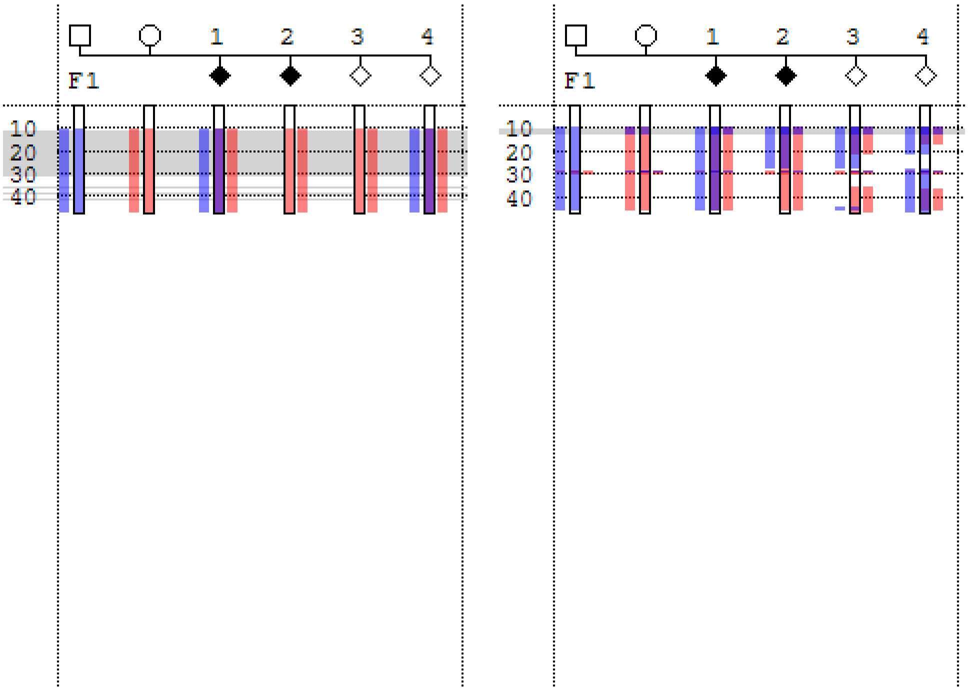

Chromosome 22

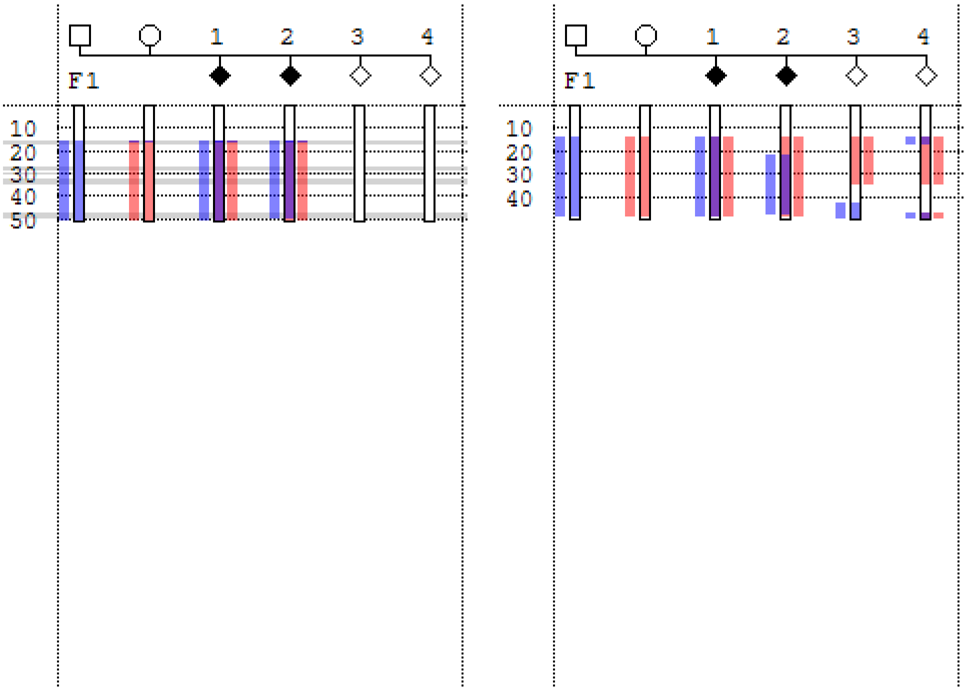

Supp. Figure S2. A comparison between exome- and microarray-derived variant data used to identify autozygous regions in consanguineous individuals. Autozygous regions identified by ***AgileVCFMapper*** using variant data derived from exomes (top three rows in each image) or Affymetrix SNP 6.0 microarrays (lower three rows in each image). The three rows in each case represent three siblings from a consanguineous family, two affected and one unaffected. In the affected siblings, autozygous regions are shaded blue; in the unaffected sibling, pink. While the overall patterns are comparable, there are differences between the two data sets. These are the result of the lower number of variants, uneven coverage and higher miscall rate of the data derived from the exomes. The centromeric and pericentromeric regions appear as large gaps in the variant coverage, and these often result in different interpretations of the extent of autozygous regions in exome and SNP microarray data.

Chromosome 1

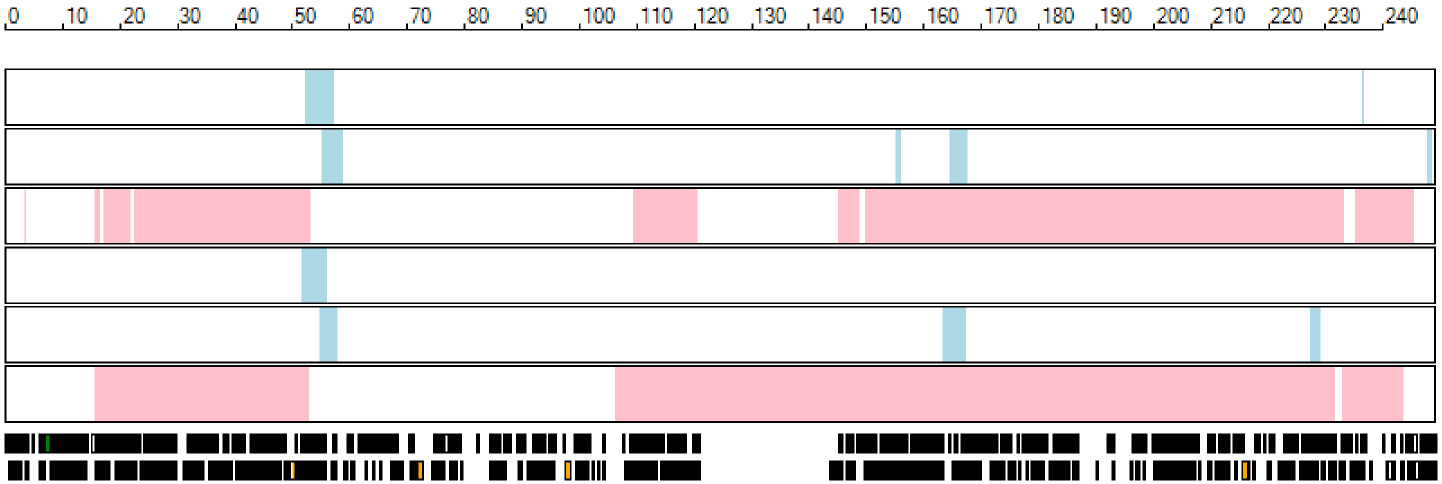

Chromosome 2

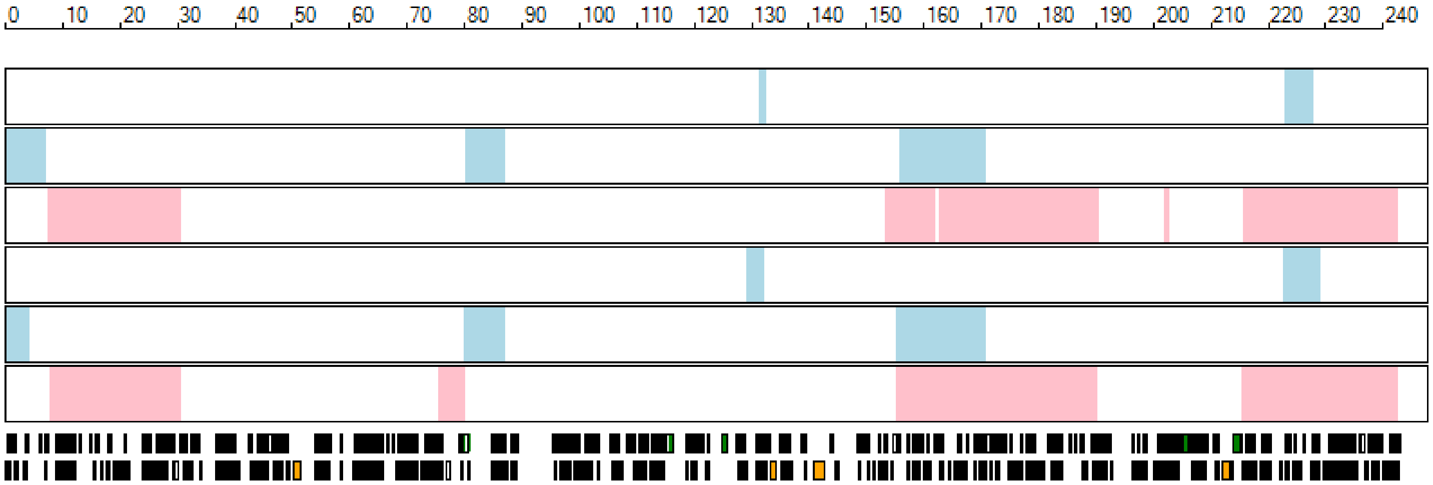

Chromosome 3

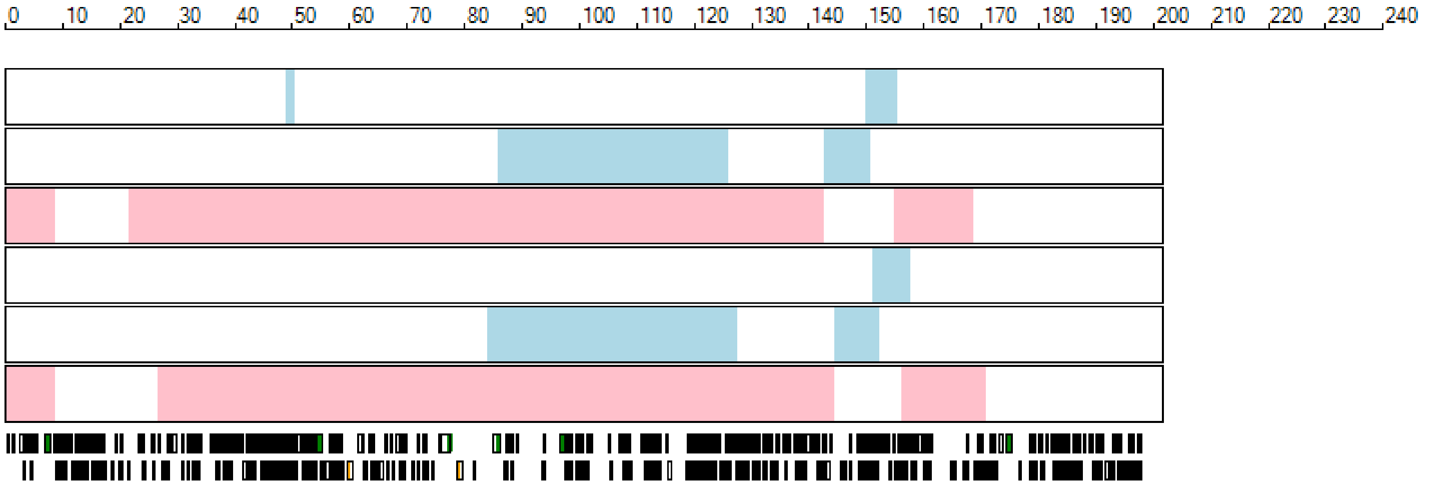

Chromosome 4

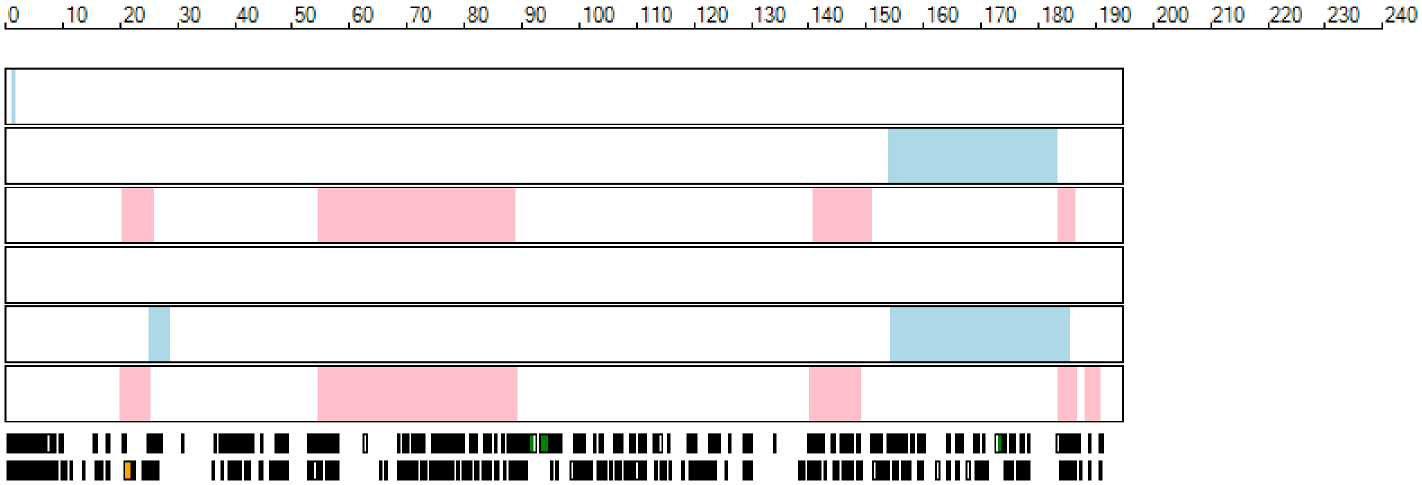

Chromosome 5

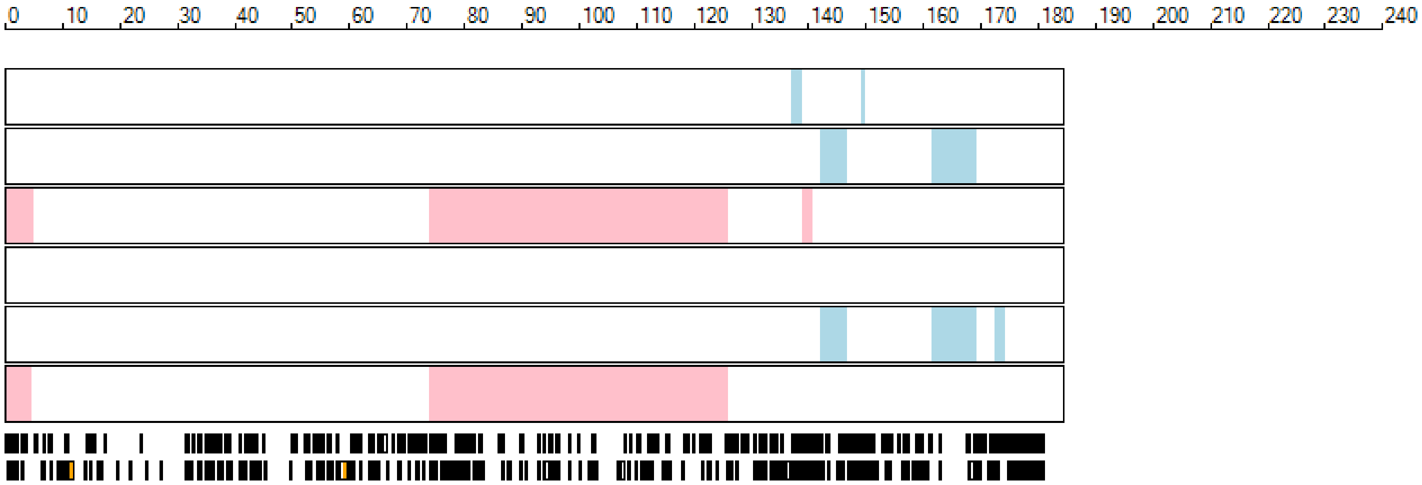

Chromosome 6

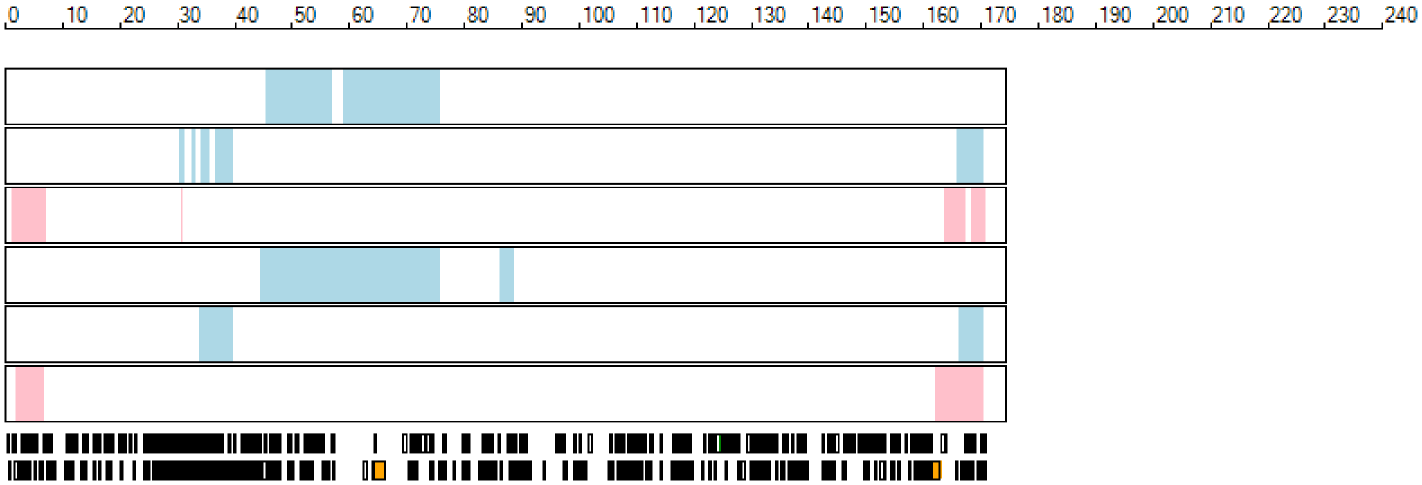

Chromosome 7

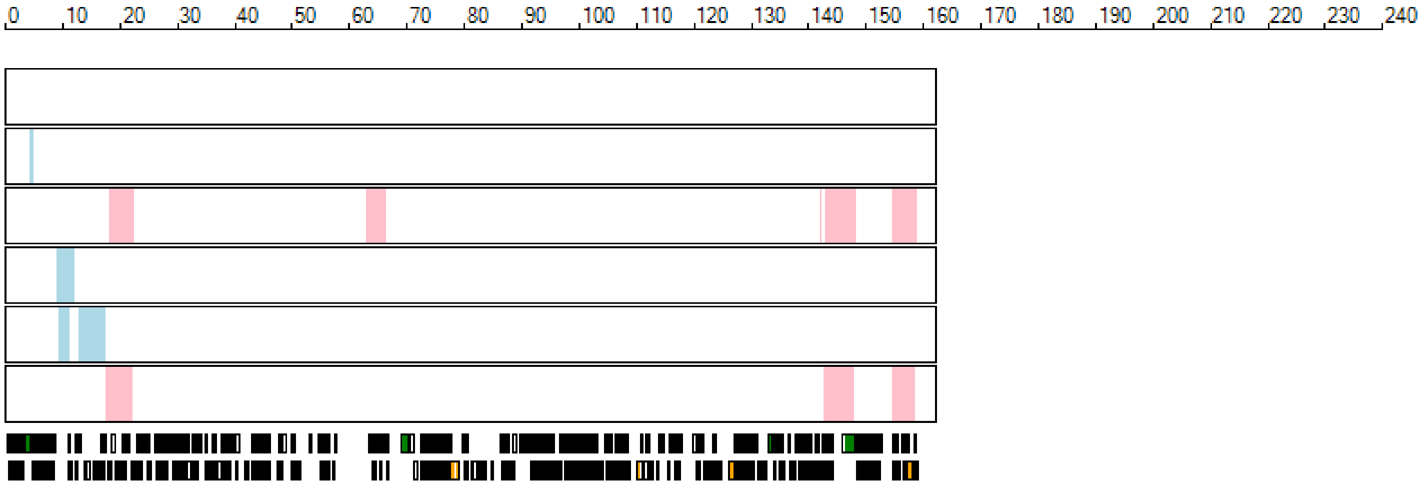

Chromosome 8

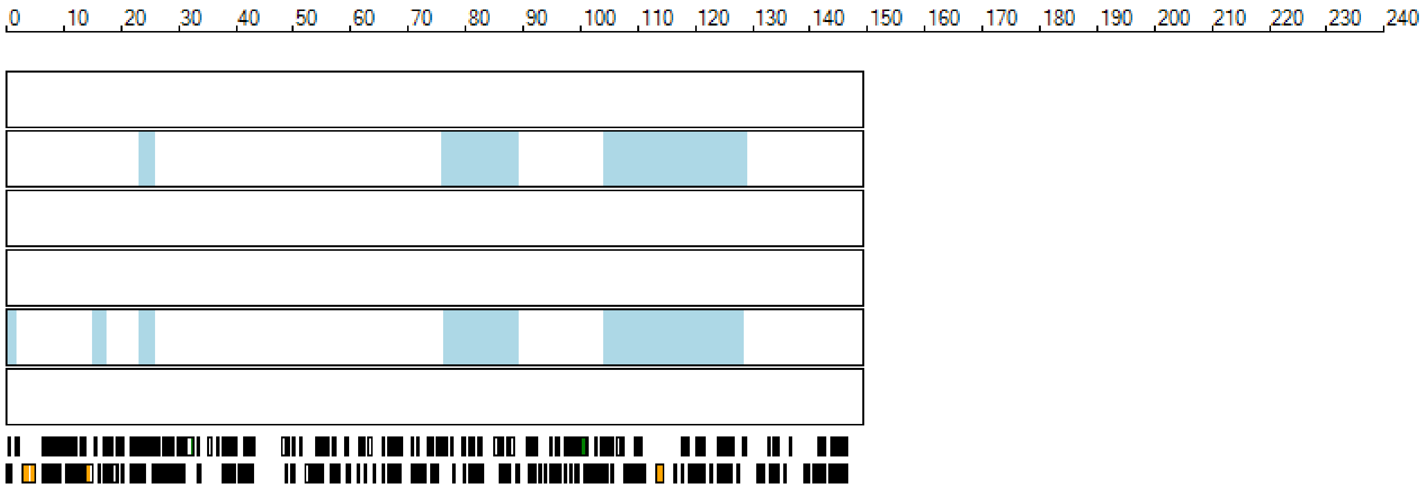

Chromosome 9

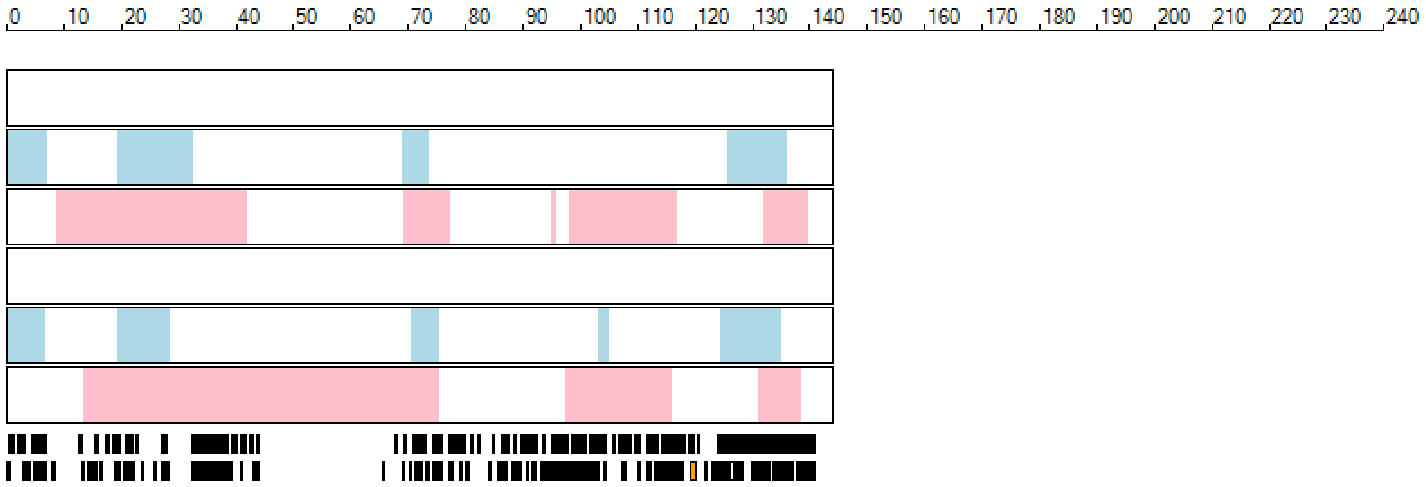

Chromosome 10

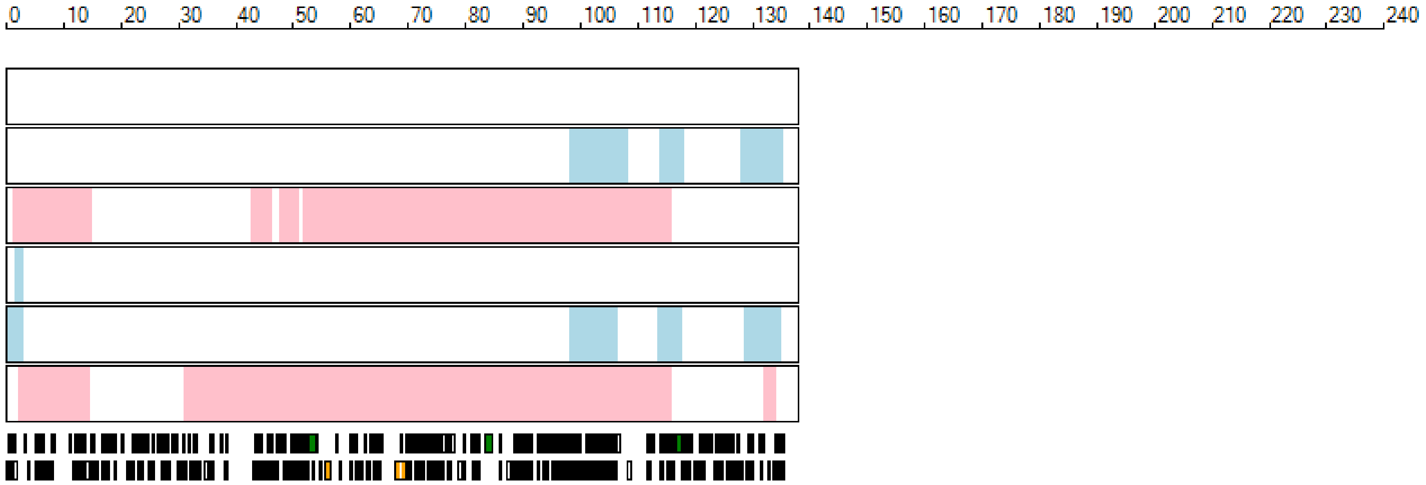

Chromosome 11

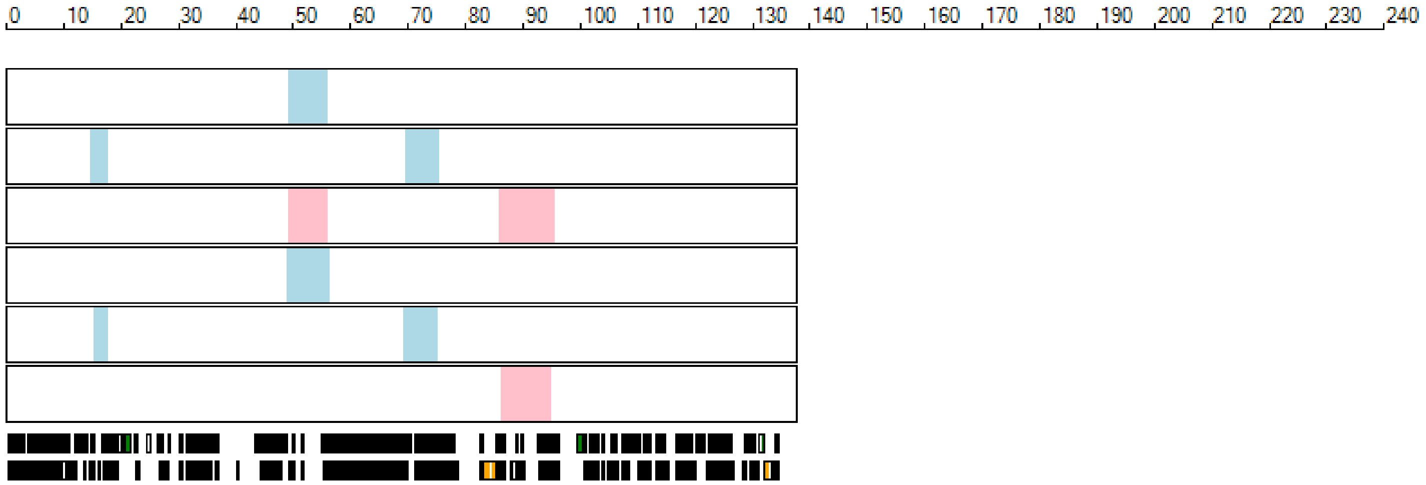

Chromosome 12

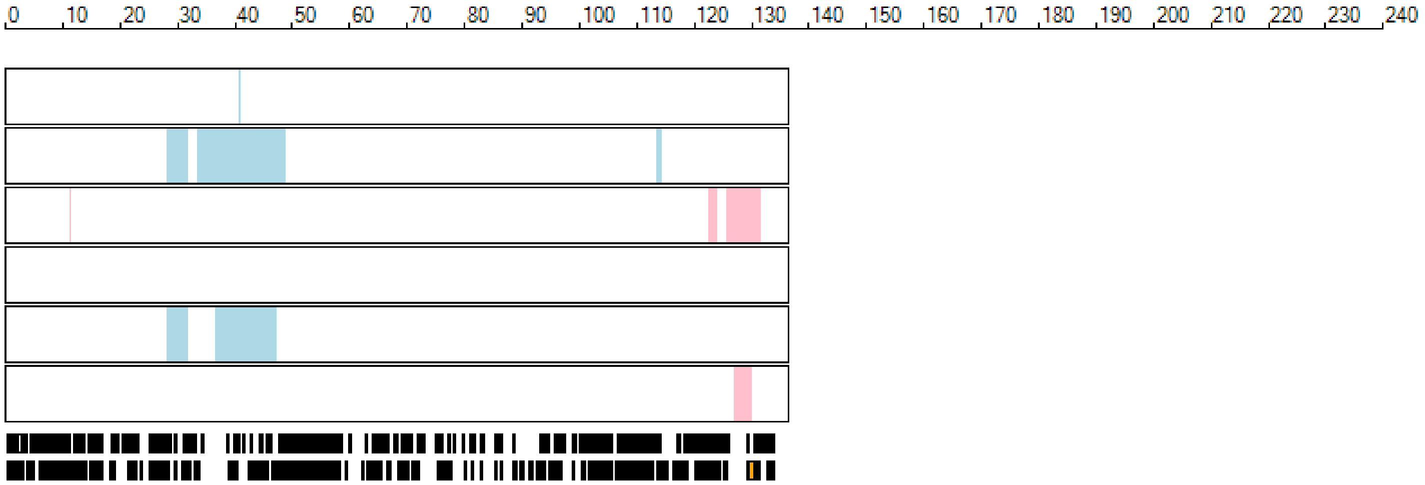

Chromosome 13

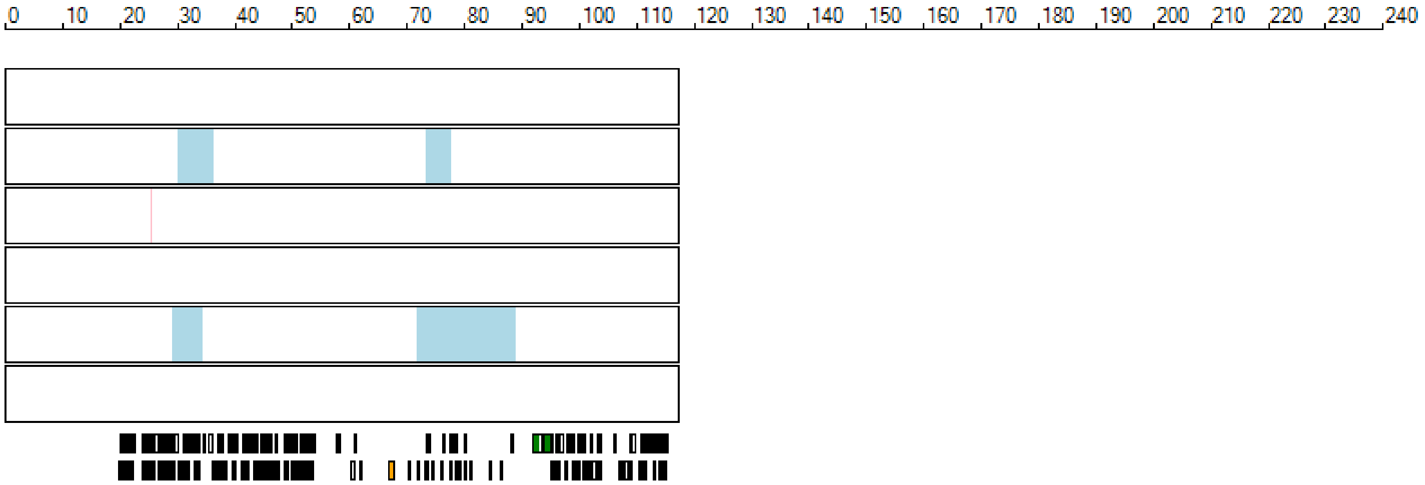

Chromosome 14

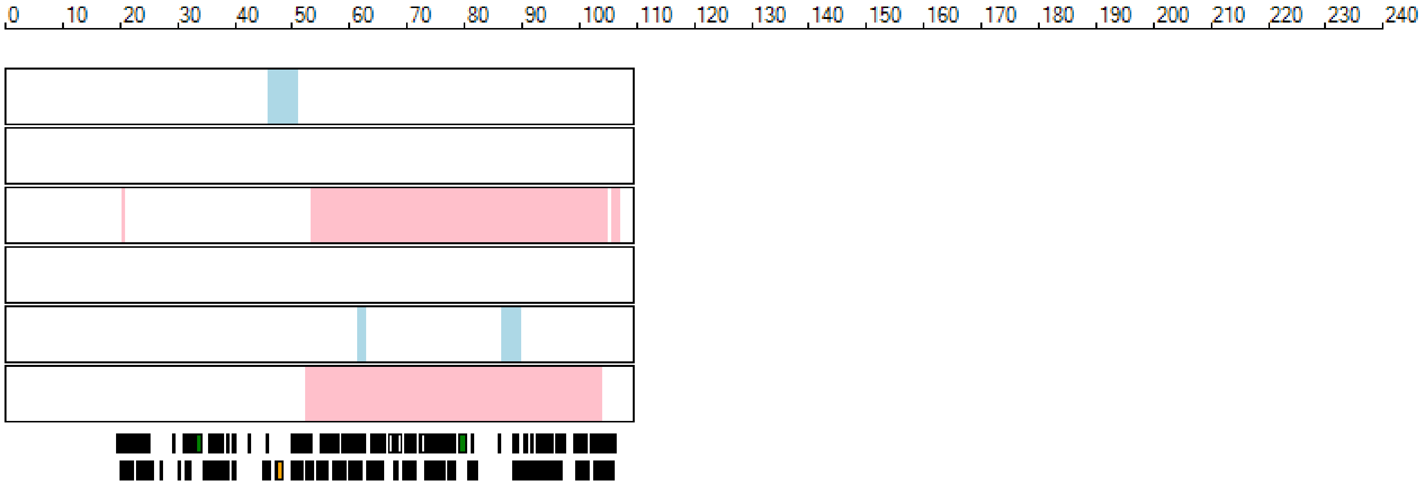

Chromosome 15

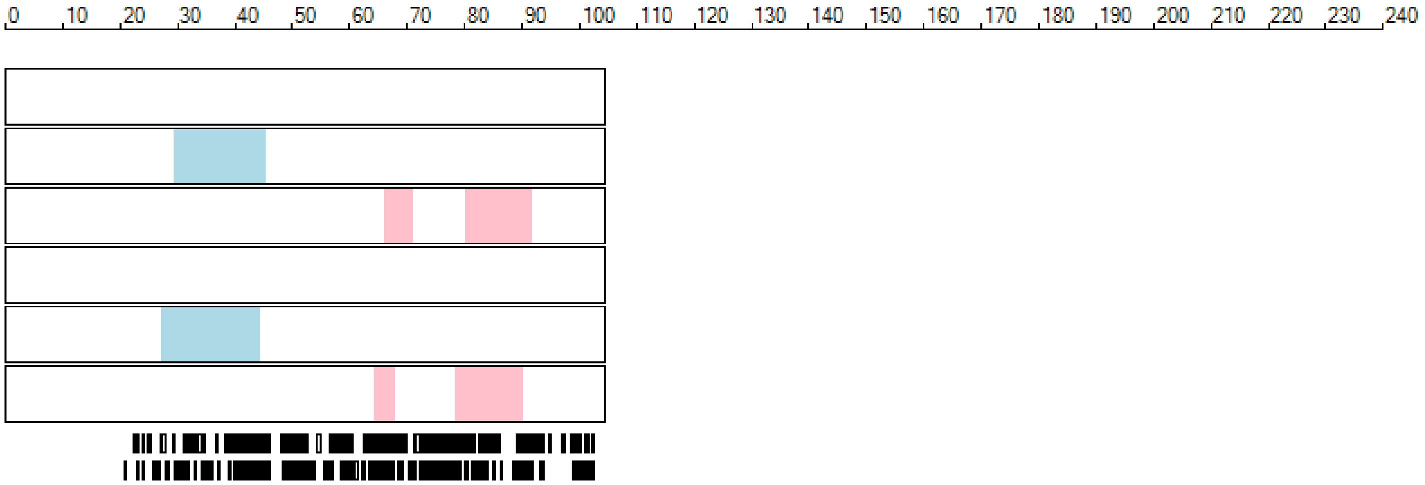

Chromosome 16

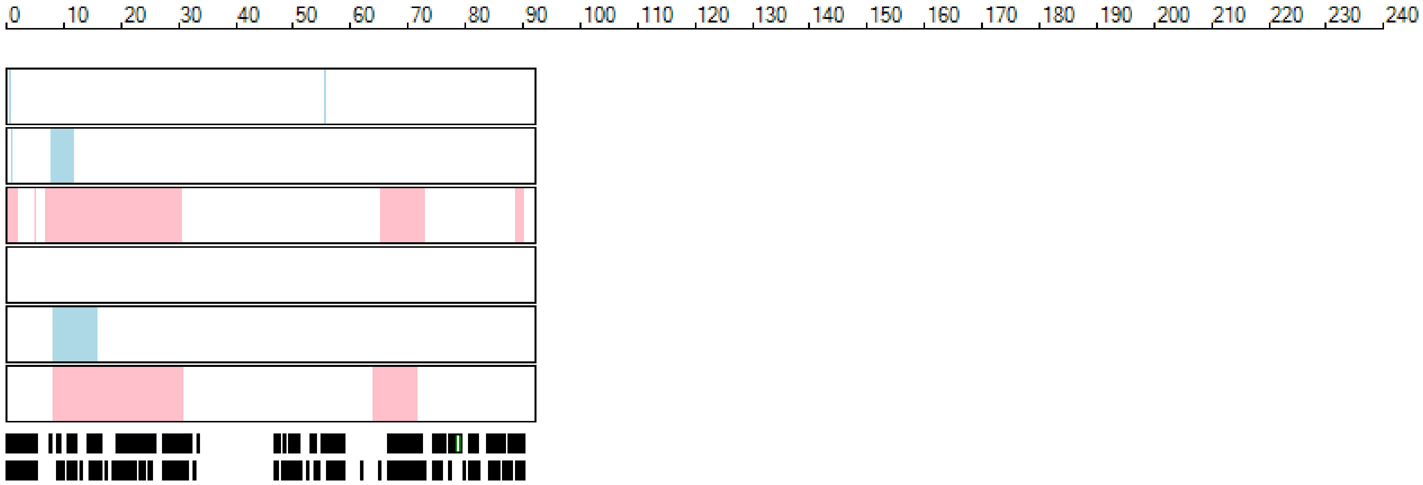

Chromosome 17

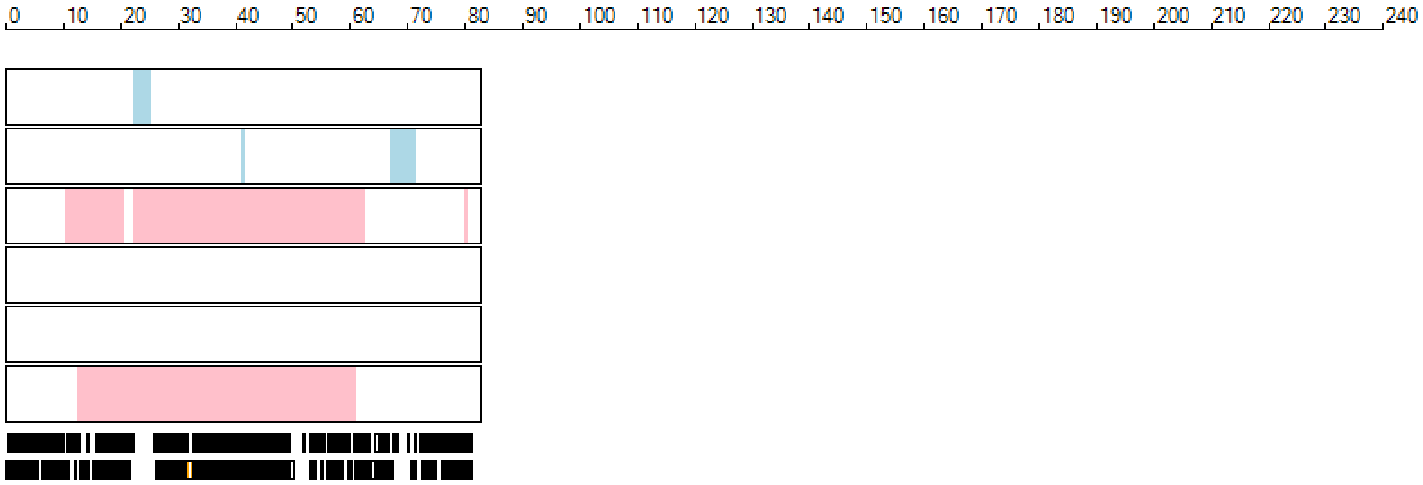

Chromosome 18

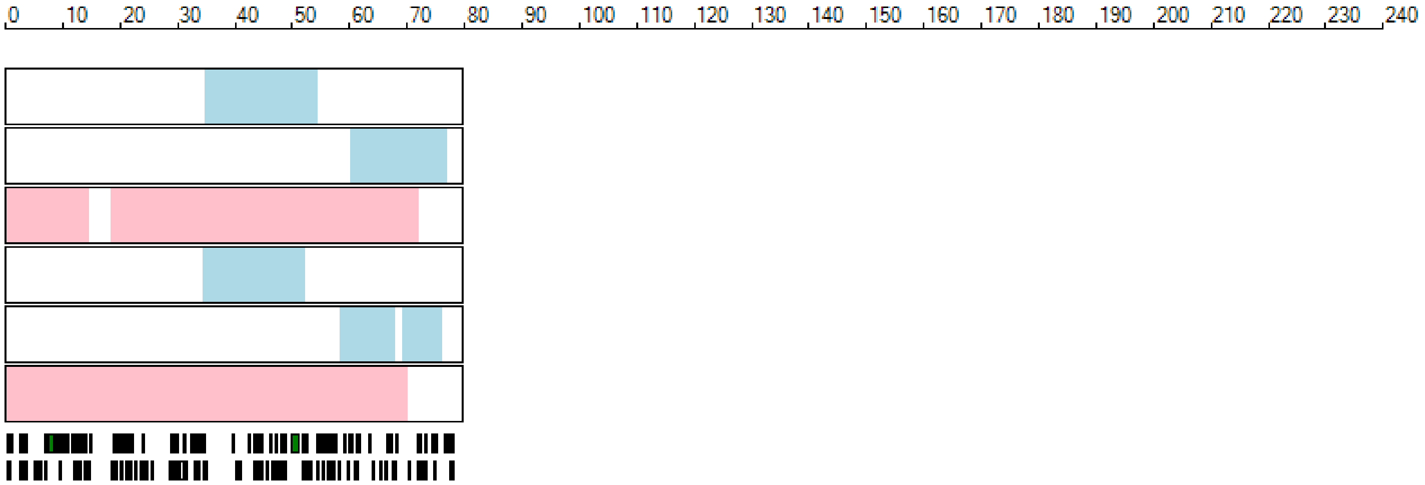

Chromosome 19

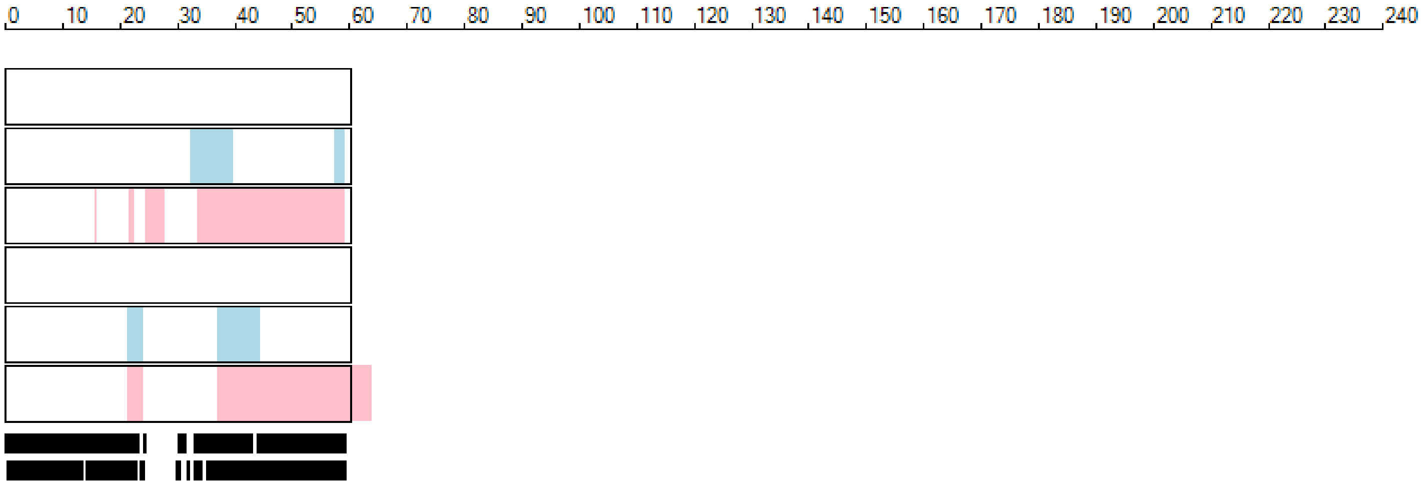

Chromosome 20

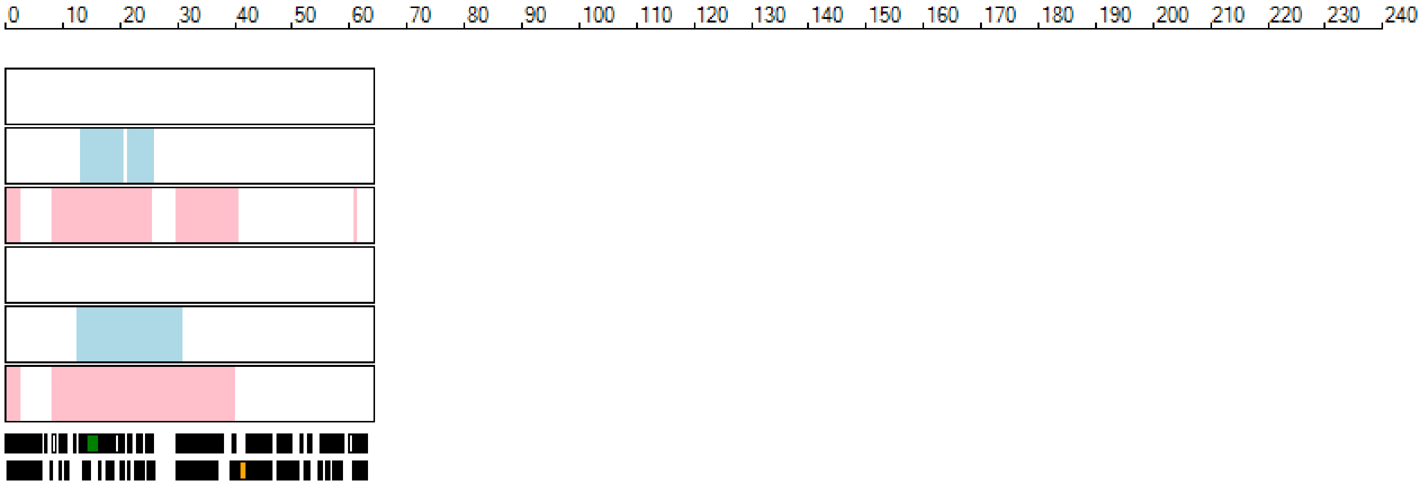

Chromosome 21

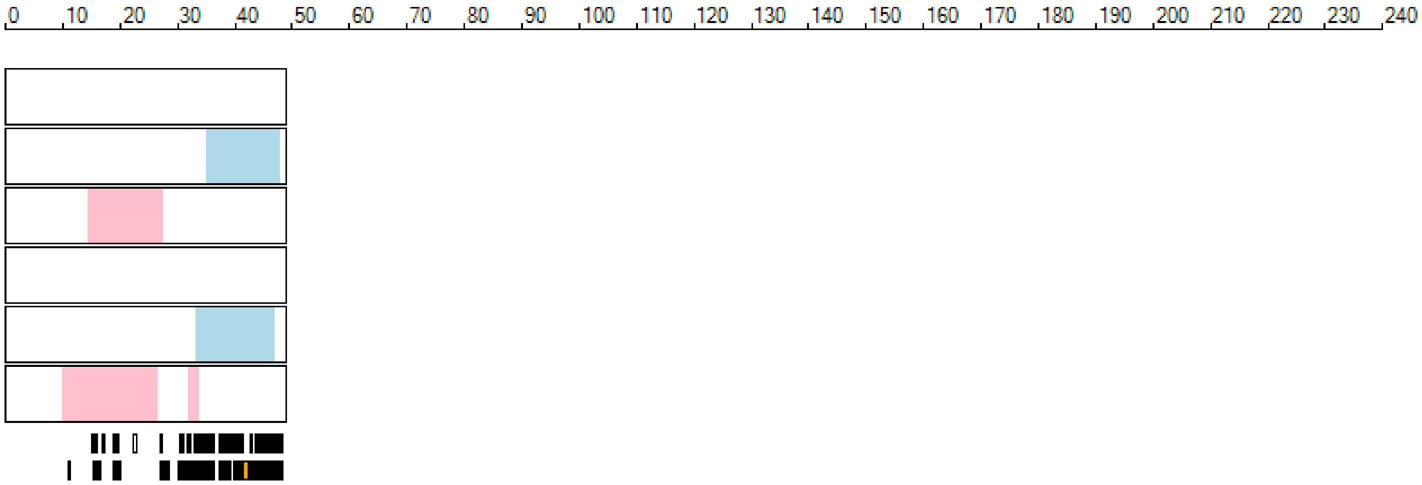

Chromosome 22

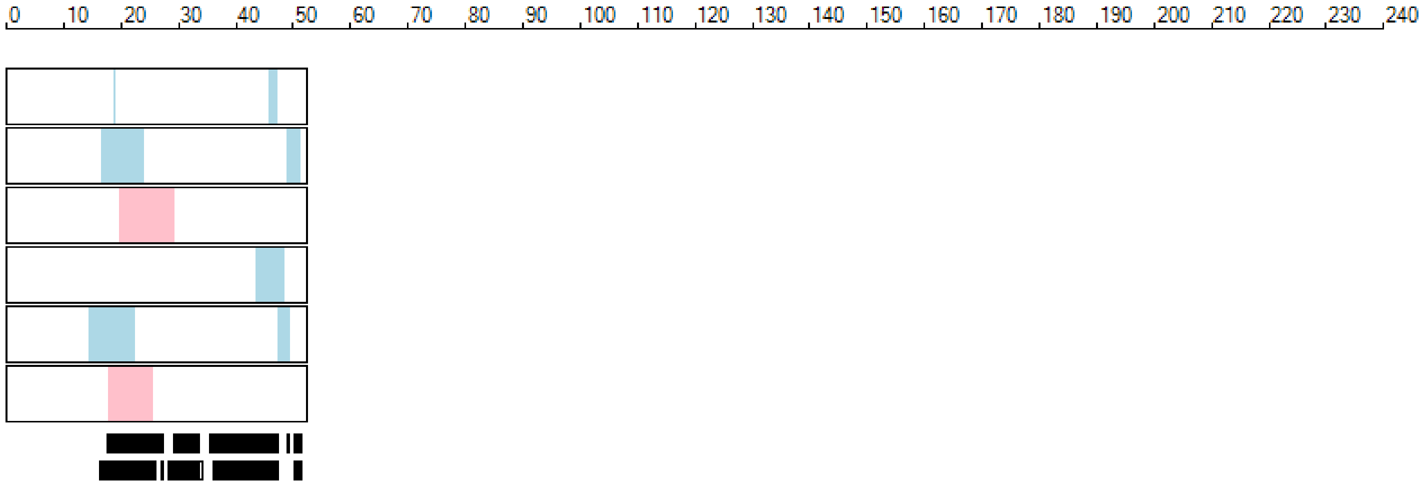

Supplement: Supplementary file 1 — Figure S1. A comparison between exome‐ and microarray‐derived variant data, displayed by Phaser (Carr et al. 2012) for the purpose of mapping a disease locus using a non‐consanguineous pedigree. Figure S2. A comparison between exome‐ and microarray‐derived variant data used to identify autozygous regions in consanguineous individuals. Table S1. The NGMS ids for the individuals in Pedigree 3 [file HUMU-36-823-s001.pdf]
